# Systematic analysis of drug combinations against Gram-positive bacteria

**DOI:** 10.1038/s41564-023-01486-9

**Published:** 2023-09-28

**Authors:** Elisabetta Cacace, Vladislav Kim, Vallo Varik, Michael Knopp, Manuela Tietgen, Amber Brauer-Nikonow, Kemal Inecik, André Mateus, Alessio Milanese, Marita Torrissen Mårli, Karin Mitosch, Joel Selkrig, Ana Rita Brochado, Oscar P. Kuipers, Morten Kjos, Georg Zeller, Mikhail M. Savitski, Stephan Göttig, Wolfgang Huber, Athanasios Typas

**Affiliations:** 1 European Molecular Biology Laboratory, Genome Biology Unit, Heidelberg, Germany; 2 Collaboration for joint PhD degree between EMBL and Heidelberg University, Faculty of Biosciences, Heidelberg, Germany; 3 Goethe University Frankfurt, University Hospital, Institute for Medical Microbiology and Infection Control, Frankfurt am Main, Germany; 4 European Molecular Biology Laboratory, Structural and Computational Biology Unit, Heidelberg, Germany; 5 Department of Biology, Institute of Microbiology, and Swiss Institute of Bioinformatics, ETH Zurich, Zurich, Switzerland; 6 Faculty of Chemistry, Biotechnology and Food Science, Norwegian University of Life Sciences, Ås, Norway; 7 Cluster of Excellence EXC 2124 Controlling Microbes to Fight Infections, University of Tübingen, Tübingen, Germany; 8 Department of Molecular Genetics, Groningen Molecular Biology and Biotechnology Institute, University of Groningen, Groningen, the Netherlands; 9 Present Address: Institute of Medical Microbiology, University Hospital of RWTH, Aachen, Germany; 10 Present Address: Interfaculty Institute of Microbiology and Infection Medicine, University of Tübingen, Tübingen, Germany

**Keywords:** Antibiotics, High-throughput screening, Antimicrobial resistance

## Abstract

Drug combinations can expand options for antibacterial therapies but have not been systematically tested in Gram-positive species. We profiled ~8,000 combinations of 65 antibacterial drugs against the model species *Bacillus subtilis* and two prominent pathogens, *Staphylococcus aureus* and *Streptococcus pneumoniae*. Thereby, we recapitulated previously known drug interactions, but also identified ten times more novel interactions in the pathogen *S. aureus*, including 150 synergies. We showed that two synergies were equally effective against multidrug-resistant *S. aureus* clinical isolates in vitro and in vivo. Interactions were largely species-specific and synergies were distinct from those of Gram-negative species, owing to cell surface and drug uptake differences. We also tested 2,728 combinations of 44 commonly prescribed non-antibiotic drugs with 62 drugs with antibacterial activity against *S. aureus* and identified numerous antagonisms that might compromise the efficacy of antimicrobial therapies. We identified even more synergies and showed that the anti-aggregant ticagrelor synergized with cationic antibiotics by modifying the surface charge of *S. aureus*. All data can be browsed in an interactive interface (https://apps.embl.de/combact/).

## Abstract (web summary)

Antibacterial combinations that are potent against Gram-positive bacteria are identified using an automated high-throughput screen.

## Main

Antibacterial agents have been used in combination for decades for different purposes: to achieve synergy (for example, sulfamethoxazole-trimethoprim), to limit resistance (for example, combinations of beta-lactams and beta-lactamase inhibitors, or antitubercular regimens) and/or to broaden the spectrum of action of anti-infective treatments (for example, empiric treatments of sepsis)^1^. With antimicrobial resistance (AMR) posing a global threat to public health, which permeates all domains of modern medicine^2,3^, the use of drug combinations to re-sensitize resistant strains has emerged as a promising means to bypass the stagnant drug discovery pipeline^4^.

Although a few antibacterial combinations are used in clinics, and screens for approved compounds as adjuvants for antibiotics have been increasingly conducted in the past decade^5–10^, the full potential of drug combinations for treating bacterial pathogens remains underexplored. This is because the combinatorial space is vast and drug interactions are rare and concentration-, drug-, time-, species- and even strain-specific^11–13^, making systematic testing necessary, yet highly demanding. As a result, drug interactions have not yet been systematically profiled in many clinically relevant bacterial species. In addition, with the increase of polypharmacy^14^, antibiotics are often prescribed in combination with other medications^15^. While pharmacokinetic interactions between antibiotics and non-antibiotic drugs are well-known for the host (for example, dependencies on drug metabolism and excretion by the liver and the kidney)^16^, they are poorly characterized at the level of bacterial physiology.

Here we used an automated platform to systematically profile drug interactions against three Gram-positive bacterial species: the pathogens *Staphylococcus aureus* and *Streptococcus pneumoniae*, two of the most prominent antibiotic-resistant bacteria^2,17^ and the model organism *Bacillus subtilis*. Compared with previous studies^7,9,18^, this vastly increased the number of drugs, concentrations and strains tested. By probing all main classes of antibiotics, we could relate interaction outcomes to bacterial structural features, cellular network architecture, as well as drug target conservation. When comparing the drug interaction networks to those of three Gram-negative pathogens obtained with a similar setup^11^, we could highlight clear differences driven by the distinct drug permeability barriers across this divide. Moreover, we profiled the interactions of antibiotics with a large panel of non-antibiotic drugs in *S. aureus* to investigate the impact of commonly administered medications on antibiotic efficacy. Thereby, we uncovered both strong synergies that proved effective against multidrug-resistant clinical *S. aureus* isolates and widespread antagonisms that could compromise the efficacy of antibiotic treatments.

## Results

### Automated high-throughput testing of drug combinations

**Fig. 1:**
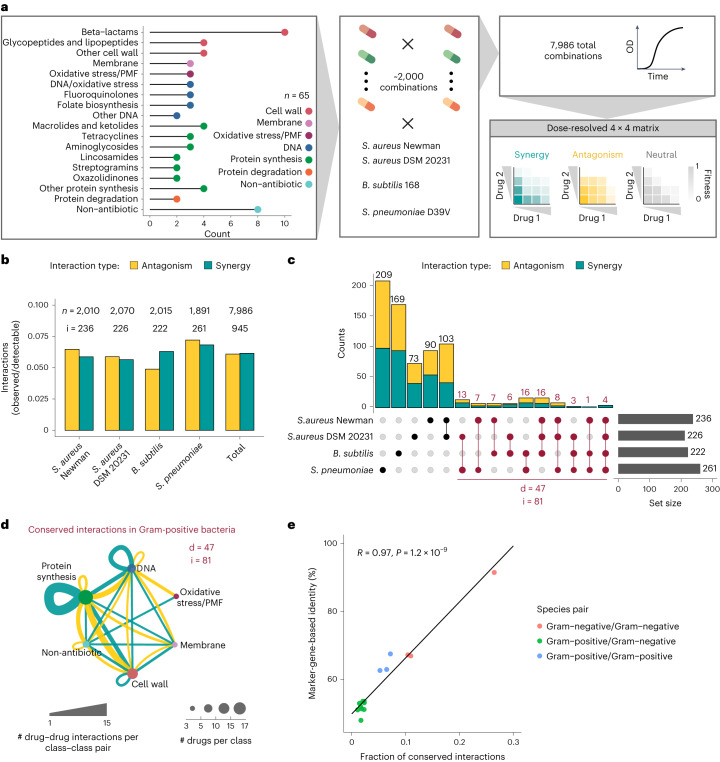
Drug–drug interactions are species-specific in Gram-positive bacteria. **a**, Schematic representation of the high-throughput screen. Pairwise combinations of 65 drugs belonging to several chemical classes and targeting different cellular processes (Supplementary Table 2) were tested at three concentrations in *S. aureus* (two strains), *B. subtilis* and *S. pneumoniae*. For each strain, 1,891–2,070 combinations were tested in broth microdilution in 384-well plates, measuring OD_595_ over time. Normalized fitness values were calculated and used to obtain 4 × 4 checkerboards and assign interactions as synergistic, antagonistic or neutral (Methods, Extended Data Fig. 1 and Supplementary Table 3). PMF, proton-motive force. **b**, Interaction abundance in each strain separately and altogether. Synergy and antagonism frequencies were obtained by dividing their absolute counts by the number of combinations for which the probed fitness space allows detection of synergy (fitness upon combination ≥0.1) or antagonism (fitness upon combination ≤0.9) discovery (Methods). Total numbers of combinations tested (*n*) and detected interactions (i) are shown for each set. **c**, Conservation of interactions among the four strains tested. All unique interactions detected in the screen (*n* = 725) were considered to calculate intersection sets between strains. The total number of interactions dependent on whether conserved or unique to each strain/species are shown. A total of 81 interactions (i), involving 47 drugs (d), are conserved across species (dark red). The total number of interactions in each strain is indicated as set size (bottom right), adding up to 945 total interactions in all strains. **d**, Network of conserved interactions between Gram-positive species. Drugs are grouped according to their targeted cellular process (Supplementary Table 2). Edge thickness is proportional to the number of drug–drug interactions for each class–class pair. Node size is proportional to the number of drugs in each class. Only drugs involved in this interaction set are considered (d = 47). Nodes are coloured according to the targeted cellular processes as in Fig. 1a. **e**, Drug interaction conservation between species recapitulates phylogeny. Pearson correlation between sequence identity (based on 40 conserved marker genes) and drug interaction conservation rate is between pairs of species tested here and previously^11^. The *P* value was obtained from a two-tailed one-sample *t*-test assessing the significance of the Pearson correlation (*H*_0_: {*t* = 0, *R* = 0}). Source data

We profiled 1,891–2,070 drug combinations in a 4 × 4 dose matrix (2-fold dilution gradient) in *S. aureus*, *S. pneumoniae* and *B. subtilis* (Fig. 1a and Supplementary Table 1). For *S. aureus*, two strains (Newman and DSM 20231) were probed to assess within-species conservation. The drug panel (*n* = 65) included antibiotics (*n* = 57) used against infections with Gram-positive bacteria, belonging to all main classes and targeting different bacterial processes, and eight other bioactive molecules, such as antifungals, drugs with human targets and food additives, depicted as non-antibiotics (Fig. 1a and Supplementary Table 2).

**Extended Data Fig. 1:**
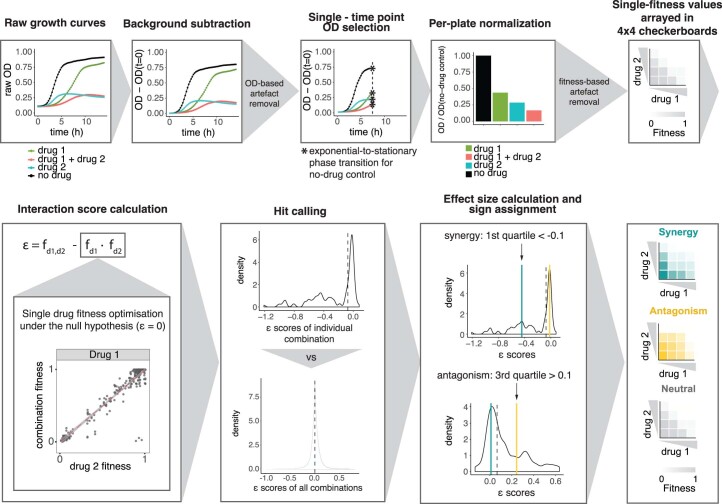
Data analysis pipeline. Raw growth curves based on measurement of OD_595nm_ over 14 h were processed as depicted. Background was removed by subtracting the OD_595nm_ at the first time point (when this was not affected by artefacts) from all the following measurements (Methods). All curves within a plate were trimmed beyond the point that the no-drug controls within the plate (6 wells) entered stationary phase. The OD_595nm_ measurement at this time-point was then normalised per plate by the robust mean (via smoothed Huber estimator^71^) of the no-drug control wells (6 per plate), resulting in fitness values (Methods) for 4 × 4 concentration checkerboards for each combination. Bliss (ε) scores were then calculated as follows: ε = *f*_d1,d2_ – *f*_d1_ * *f*_d2_, where *f*_d1,d2_ corresponds to the observed fitness in the presence of the drug combination, and *f*_d1_ and *f*_d2_ correspond to the fitness in the presence of each single drug at the concentration in combination. Single-drug fitness values were estimated from drug-combination fitness by minimizing the sum of residuals squared of the Bliss independence model (Methods, [Eq. 2]). Interactions fulfil two criteria: (i) FDR < 0.05, after applying a resampling procedure (10,000 repetitions of a two-sided Wilcoxon rank-sum test) to compare the ε distribution of each combination tested to the overall ε distribution; and (ii) a quartile-based effect size threshold examining the ε distribution of each combination, with synergies assigned if first quartile (green line) < −0.1 and antagonisms if third quartile > 0.1 (yellow line). Source data

**Extended Data Fig. 2:**
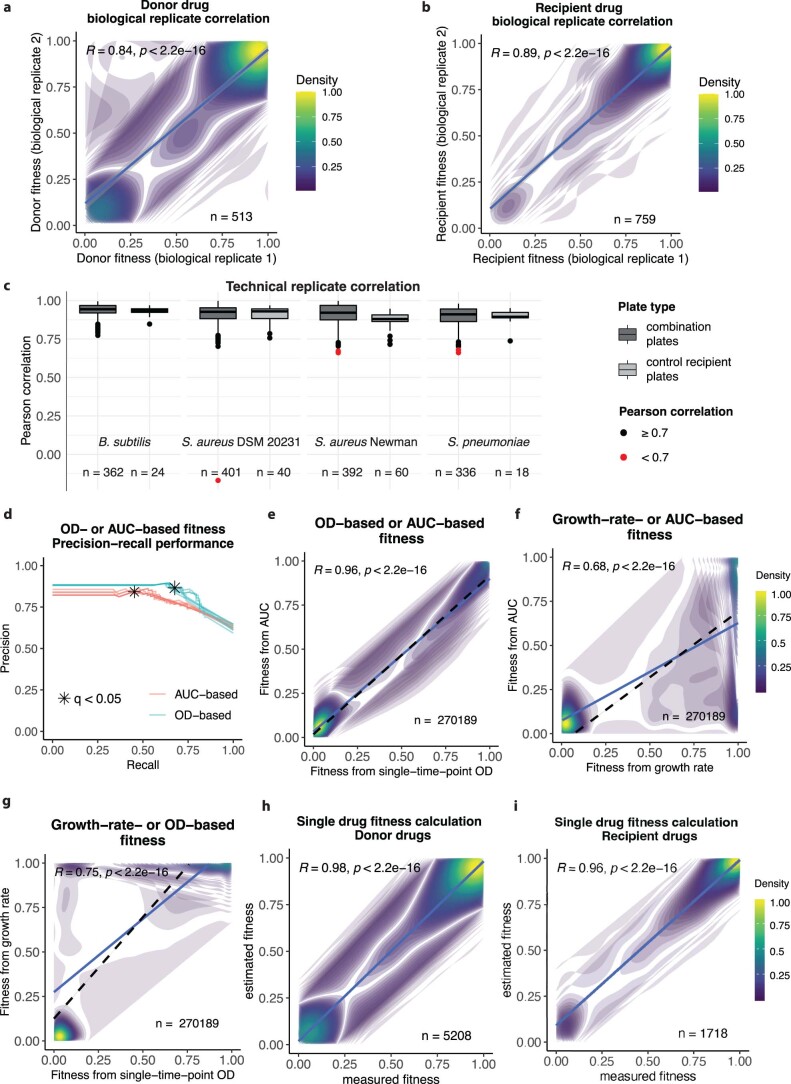
Quality control of the main interaction screen and assessment of fitness calculation methods. **a-b**, Donor (**a**) and recipient (**b**) drug fitness correlation between biological replicates. Pearson correlation (R = 0.84 and 0.89, respectively, p < 2.2e-16) is calculated between biological replicates, corresponding to different experimental runs/batches. P-values are obtained from a two-tailed one-sample *t*-test assessing the significance of the Pearson correlation (${H}_{0}:\{t=0,{R}=0$}). **c**, Technical replicate correlation. Pearson correlation is calculated between replicate wells within the same plate for combination plates (where donor drugs were added) and control recipient plates (where no donor drug was added), for the four strains screened. Plates for which technical plate correlation was < 0.7 (red) were removed from the data. Box limits correspond to first and third quartiles, with the median marked, and whiskers to the most extreme data points up to 1.5 times the IQR. **d**, Performance of endpoint OD- and AUC-based measurements against the benchmarking set. Precision-recall curves are shown for q-value intervals increasing by 0.01. Curves highlighted correspond to the effect-size cut-off selected for the screen (interaction score = |0.1|). The significance cut-off (FDR < 0.05) is marked. **e-g**, Endpoint-OD- and AUC-based fitness values for all strains are highly correlated (**e**; Pearson correlation, R = 0.96, p < 2.2e-16, obtained from a two-tailed one-sample *t*-test assessing the significance of the Pearson correlation, n = 270189), whereas fitness values only based on growth rate correlated worse with either AUC- (**f**) or OD-based (**g**) fitness values (Pearson correlation, R = 0.68 and 0.75, respectively, p < 2.2e-16, obtained as in **e**, n = 270189). **h-i**, Comparison between estimated and experimentally measured single-drug fitness for donor (**h**) (Pearson correlation, R = 0.98, p < 2.2e-16, obtained as in **e**, n = 5208) and recipient (**i**) drugs (Pearson correlation, R = 0.96, p < 2.2e-16, obtained as in **e**, n = 1718). Source data

We measured growth in a broth microdilution format in microtitre plates using optical density at 595 nm (OD_595_) as a readout. Media and shaking conditions were different for each species (Methods). Drug concentrations were tailored after measuring minimal inhibitory concentrations (MICs) and the drug concentrations causing 50% growth inhibition (IC_50s_) for each drug in the four strains, with the highest concentration corresponding to the MIC in most cases, and the intermediate and lowest concentration corresponding to half and a quarter of the highest concentration, respectively (Methods and Supplementary Table 2). We derived fitness values in the presence of single drugs and drug combinations, dividing single-timepoint OD_595_ values upon drug treatment by the corresponding values of no-drug controls at the same timepoint. This timepoint was different for each strain and corresponded to the entry to stationary phase in the absence of drugs, allowing us to capture drug effects on both growth rate and yield (Methods and Extended Data Fig. 1). We conducted all experiments in biological (that is, different overnight cultures) and technical (that is, inoculated wells in the same plate) duplicates, achieving high replicate correlations (average Pearson correlation of 0.84–0.89 for biological (Extended Data Fig. 2a,b) and 0.91 for technical replicates (Extended Data Fig. 2c)). Fitness based on single-timepoint OD_595_ and area under the growth-curve (AUC) led to very similar results, with the former being more accurate (Extended Data Fig. 2d,e). In contrast, fitness values based only on growth rate had a lower correlation to the two other metrics, overestimating fitness for some drug treatments (Extended Data Fig. 2f,g). For single-drug fitness, we used estimated values derived from the combination experiments, as they were concordant with experimental measurements and were derived from more data points (Extended Data Fig. 2h,i and Methods). From the 4 × 4 concentration matrices of fitness values, we calculated interaction scores using the Bliss interaction model^19^ (Methods and Extended Data Fig. 1). A single effect-size value was derived from the distribution of interaction scores for each drug pair (at least 72 scores, including all replicates of individual concentration combinations). The first and third quartile values of this distribution were taken as effect-size values for synergies and antagonisms, respectively, with negative values corresponding to synergies and positive values to antagonisms (Methods, Extended Data Fig. 1 and Supplementary Table 3)^11^. All interaction data are available for browsing in a user-friendly interface (https://apps.embl.de/combact/).

**Extended Data Fig. 3:**
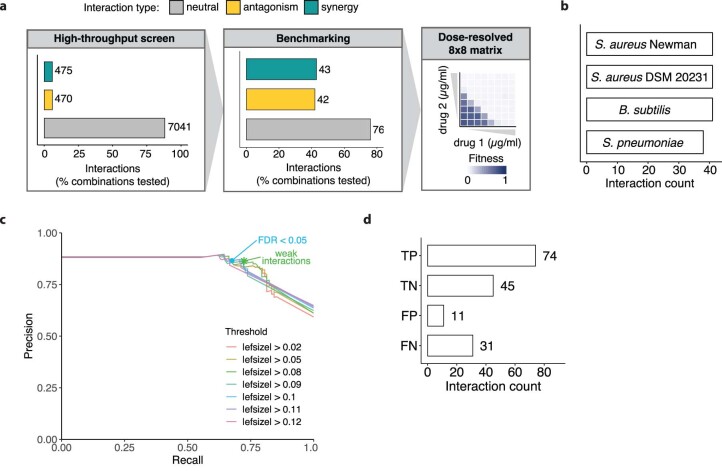
Screen benchmarking. **a**, 161 drug combinations were selected for benchmarking, including hits and neutral interactions, and tested in extended concentration checkerboards (8 x 8). Fitness values and interaction scores were calculated as in the high-throughput screen (Methods). **b**, Combinations were selected to equally represent the four strains tested. **c**, Screen precision and recall against the benchmarking set are assessed for different effect-size thresholds. Precision-recall curves are shown for FDR intervals ranging from 0 to 1, increasing by 0.005. The chosen significance value for the screen (FDR < 0.05) is highlighted for the effect-size curve (|0.1|) providing best precision and recall. The addition of weak interactions (effect-size threshold |0.08|, Methods) increases slightly the recall. |efsize| = effect size. **d**, True-positive (TP), true-negative (TN), false-positive (FP) and false-negative (FN) abundance in the benchmarking set for optimal thresholds. As for most screens, conservative cutoffs for interactions minimize FPs with a cost on the number of FNs. Source data

To calibrate hit scoring, as well as to assess the high-throughput screen data quality, we benchmarked the screen data against a validation set of 161 combinations (2% of screened combinations), equally representing the four strains probed. These combinations were tested in the same growth conditions as our high-throughput screen, but over a highly resolved dose space (8 × 8 matrix) of linearly spaced concentration gradients (Methods, Extended Data Fig. 3a,b and Supplementary Table 4). The precision–recall curves were comparable to the previous Gram-negative screen^11^, with highest precision (0.87) and recall (0.68) observed for a threshold on absolute effect size of >0.1 and on Benjamini–Hochberg adjusted *P* value of <0.05 (resampling procedure with 10,000 repetitions for each combination tested, comparison with resampled Bliss scores using Wilcoxon rank-sum test in each iteration) (Methods and Extended Data Fig. 3c). The lower recall was linked to our increased ability to detect mild interactions in the extended 8 × 8 concentration matrices of the benchmarking dataset and is a direct trade-off for the high precision cut-off we set in the screen to limit false positives. We were able to increase the recall to 0.72 by relaxing the effect-size thresholds for interactions found in both *S. aureus* strains, using within-species conservation as an additional parameter to confirm interactions^11^ (Methods, Extended Data Fig. 3c,d and Supplementary Table 3).

**Extended Data Fig. 4:**
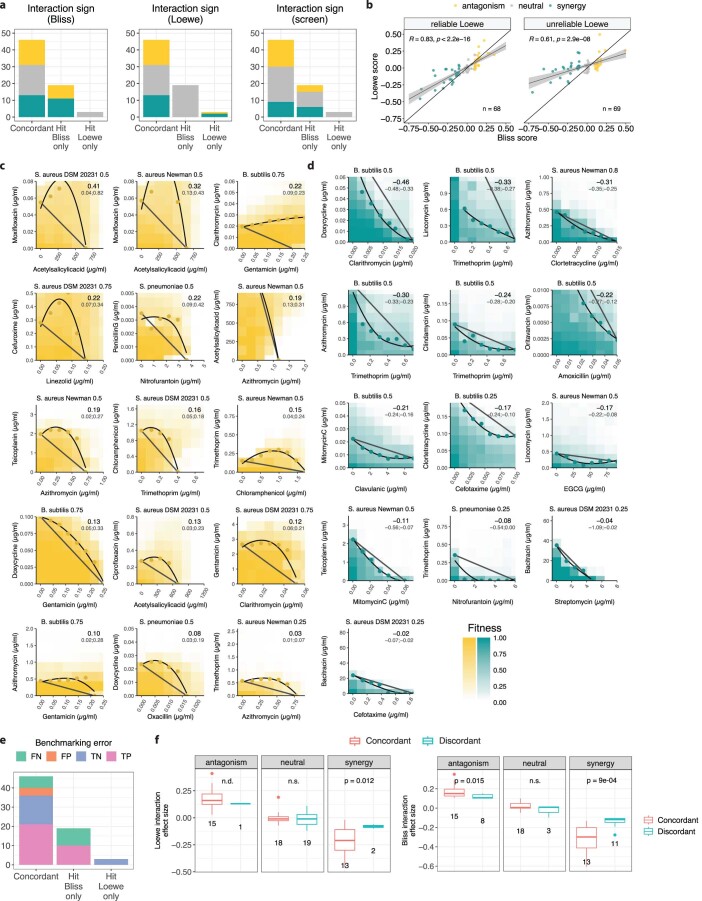
Interactions according to the Loewe model. **a**, High concordance when assessing interactions with the Loewe or Bliss model for the benchmarking set (8x8 checkerboards). Comparison results are shown only for drug pairs which both models could be reliably applied (n = 68; Methods, Supplementary Table 2). Interaction sign is shown as calculated according to the Bliss model (left), the Loewe model (middle), and the original screen (right). **b**, Pearson correlation between interaction scores calculated according to the Loewe and Bliss models for the 68 interactions for which both models could be reliably calculated (left) or the 69 for which Loewe was unreliable (right, see also Methods). Interactions are color-coded according to the Bliss model sign. P-values are obtained from a two-tailed one-sample *t*-test assessing the significance of the Pearson correlation (${H}_{0}:\{t=0$, $R=0$}). **c-d**, Synergies (**c**) and antagonisms (**d**) identified by both the Loewe and Bliss models depicted as 8x8 checkerboards with overlaid lines connecting points of equal growth inhibition, representing deviations from Loewe additivity (straight line). The fitness value (top, next to the strain) chosen for the overlaid lines, and the interaction effect size (top-right, above) as a fitness difference from additivity with its 95% confidence interval (top-right, below) according to the Loewe model, are indicated for each checkerboard. Each checkerboard is the average of at least two biological replicates, color-coded according to their Bliss-model sign. **e**, Relationship between interactions according to Loewe and Bliss models and screen benchmarking analysis according to Bliss model. TP, true positive; TN, true negative; FP, false positive; FN, false negative. **f**, Distribution of interaction effect size according to the Loewe (left) and Bliss (right) models for concordant or discordant interactions across the two models (using interactions for which Loewe can be reliably applied; n = 68). Interaction effect size according to Loewe model is estimated using the meanR statistic^72^ (Methods). Interaction effect size according to Bliss model is calculated as in the screen. Sample size of each set is indicated below each boxplot; a two-sided Welch’s *t*-test was used to determine significance – n.d. = not determined (not enough data points); n.s. = not significant. Box plots are depicted as in Extended data Fig. 2c. Source data

Although the Loewe interaction model^20^ was inadequate for the main screen (Methods), the extended concentration space probed in the benchmarking set allowed us to assess interactions also using this model. After excluding drug pairs for which the Loewe interaction model could not be used *(n* = 24; single drugs had no inhibitory effect) or was unreliable (*n* = 69; Methods and Supplementary Information), we found that the two models mostly agreed in assessing interactions (*n* = 46/68, Extended Data Fig. 4a–d and Supplementary Information). Importantly, the overall interaction scores of two models were significantly correlated, even for drug pairs for which the Loewe model was deemed unreliable (Extended Data Fig. 4b), and was considerably higher than in previous reports^21,22^. Only three interactions were captured just by the Loewe model (Supplementary Information), none of which were detected in the screen (Extended Data Fig. 4e). In contrast, out of the 19 interactions captured only by the Bliss model, 15 were concordant with the Loewe model in the sign of interaction but were missed due to the arbitrary confidence threshold (Methods and Supplementary Information). In agreement with arbitrary confidence thresholds driving the residual disagreement between the two models, concordant interactions were overall stronger in both models (Extended Data Fig. 4f,g and Supplementary Table 5).

### Drug interactions are rare and species-specific

Antagonisms and synergies were detected to be equally prevalent across the three species, accounting for ~12% of all combinations tested (Fig. 1b). This interaction rate, corrected by our ability to detect synergies or antagonisms based on the concentration space probed for each combination (Methods), is lower and less skewed towards antagonisms as compared with Gram-negative species (15% altogether considering *E. coli*, *S*. Typhimurium and *P. aeruginosa*)^11^. This could be due to technical (drug or strain selection biases, testing one strain in *B. subtilis* and *S. pneumoniae*, which prevents the use of within-species conservation to retrieve more interactions^11^) or biological reasons (Gram-positive bacteria having a lower drug permeability bottleneck than Gram-negative bacteria and hence less antagonisms; see also Discussion).

Species-specificity of drug interactions has long been assumed^23^ and recently systematically demonstrated for Gram-negative species, with 30% of detected interactions shared between at least two of the three species tested and 5% conserved in all three species (*E. coli*, *S*. Typhimurium, *P. aeruginosa*)^11^. In Gram-positive species, we observed an even lower conservation rate (Fig. 1c), with only 81 out of 725 unique interactions (11.2%) conserved in at least two species (Fig. 1d). Some 29 interactions were conserved in all three species (4%) (Supplementary Table 3). We reasoned that the lower interspecies conservation in our screen could be driven by the strain and species selection in the two screens. For Gram-negative species, two closely related *Enterobacteriaceae*, *E. coli* and *S*. Typhimurium, exhibited the highest overlap of interactions^11^, but the interaction conservation rate of either of these two species with *P. aeruginosa* is similar to the cross-species conservation rates we detected for Gram-positive bacteria. Indeed, when we compared the interaction conservation rate and genome sequence percentage identity (based on 40 universal single-copy marker genes^24^), the two were significantly correlated (Methods and Fig.1e).

**Extended Data Fig. 5:**
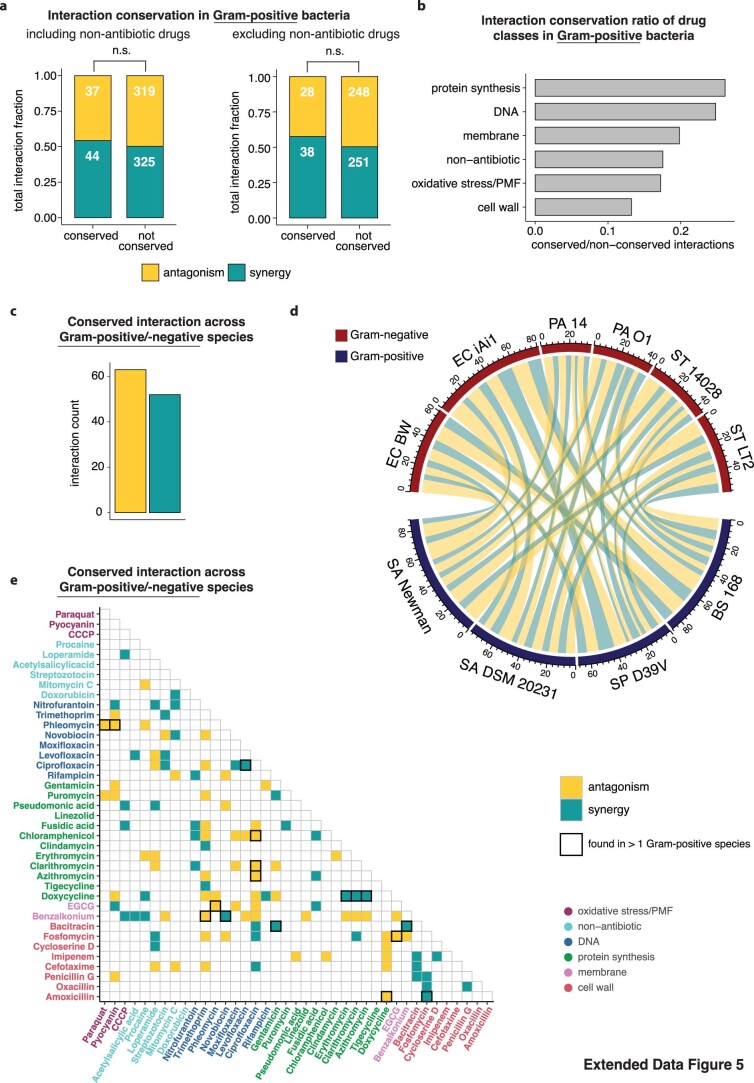
Interaction conservation within Gram-positive species and across the Gram-positive/-negative divide. **a**, There is no significant difference between synergy and antagonism prevalence among conserved and non-conserved interactions, regardless of whether non-antibiotic drugs, whose targets are multiple or unknown, are considered (p = 0.592, χ2 test) or not (p = 0.327, χ2 test). Only interactions conserved across at least two species are considered (n = 81, Fig. 1c). **b**, Drugs targeting more conserved cellular processes tend to have more conserved interactions. Interaction conservation ratio for each drug class across species is calculated as the ratio between conserved and non-conserved interactions. **c**, Synergy and antagonism abundance of unique interactions shared by at least one Gram-negative and Gram-positive strain – edges are colour-coded according to whether interaction is synergistic (green) or antagonistic (yellow). **d**, Conserved interactions across at least one Gram-negative and Gram-positive strain. EC, E.coli; PA, P. aeruginosa; ST, S. Typhimurium; SA, S. aureus; SP, S. pneumoniae; BS, B. subtilis. **e**, Heatmap of conserved interactions across Gram-positive and Gram-negative bacteria. Interactions that are also conserved across multiple Gram-positive species are highlighted. Source data

In contrast to Gram-negative bacteria^11^, we could not observe a significant enrichment for synergies among conserved interactions, even after removing non-antibiotic drugs for which intracellular targets and their conservation are unknown (Extended Data Fig. 5a). Conserved synergies were mostly driven by drugs targeting the same essential and highly conserved cellular processes, such as DNA biosynthesis and translation (Fig. 1d and Extended Data Fig. 5b). Some of these interactions, such as the synergy between macrolides and tetracyclines or between quinolones of different generations, have been observed before in Gram-negative species^11,25^, pointing towards conserved relationships between the targets of these compounds. Similarly, the broad antagonism between drugs targeting DNA and protein synthesis (Fig. 1d) is conserved in Gram-negative bacteria and is due to the alleviation of protein–DNA imbalance after treatment with any of the two antibiotics alone^26^. Overall, we detected 52 synergies and 66 antagonisms shared across the Gram-positive/-negative divide (Extended Data Fig. 5c–e and Supplementary Table 6).

### Numerous previously unknown drug synergies for *S. aureus*

**Fig. 2:**
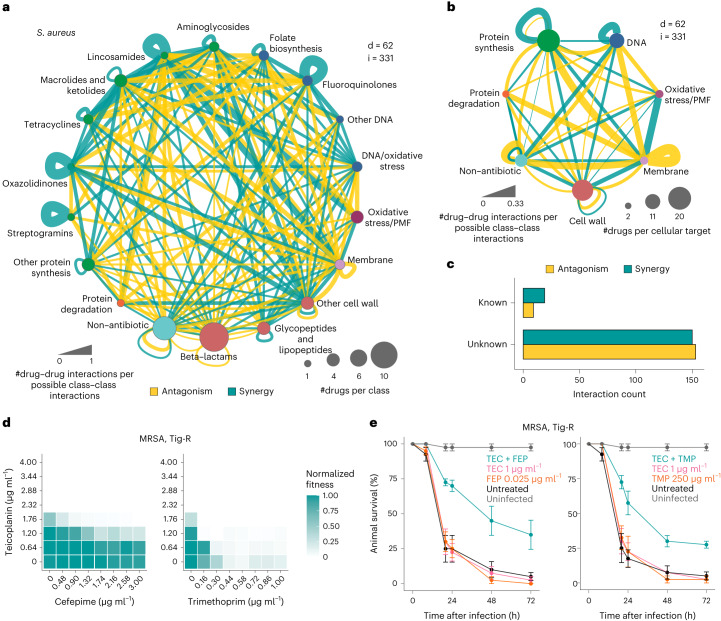
Novel synergies against *S. aureus* clinical isolates. **a**,**b**, Drug interaction networks in *S. aureus*, with drugs grouped according to their class (**a**) or targeted cellular process (**b**). Unique interactions across both strains tested are considered (i = 331). Edge thickness represents the proportion of interactions for each node pair, considering all possible interactions given the number of drugs in each node. Nodes depict the drug class (**a**) or the targeted cellular process (**b**), and size is proportional to the number of drugs in the represented class/process. Only interacting drugs are considered (d = 62). Synergies, antagonisms and nodes are coloured according to Fig. 1. **c**, Novel and previously reported interactions detected in *S. aureus*. Interactions are considered known if reported in any *S. aureus* strain (Supplementary Table 7). **d**,**e**, Identified synergies are effective against MRSA clinical isolates in vitro (**d**) and in vivo in the *G. mellonella* infection model (**e**). Teicoplanin (TEC) synergies with cefepime (FEP) and trimethoprim (TMP) were validated against a tigecycline-resistant MRSA clinical isolate (Supplementary Table 1) in 8 × 8 broth microdilution checkerboards (**d**) and in the *G. mellonella* infection model (**e**). For checkerboards, the median fitness (OD_595_ at 7.5 h normalized by no-drug controls) across two biological replicates is shown (Supplementary Information). For *G. mellonella* experiments, larvae were infected with the same MRSA isolate and treated with single drugs or combinations. The percentage of surviving larvae after treatment and in the untreated controls was monitored over time. Uninfected and untreated (vehicle only) controls are shown. Drugs were tested in combination at the same concentration indicated for each drug. Data indicate mean ± s.e. (*n* = 10 for each condition, three independent experiments). Source data

**Extended Data Fig. 6:**
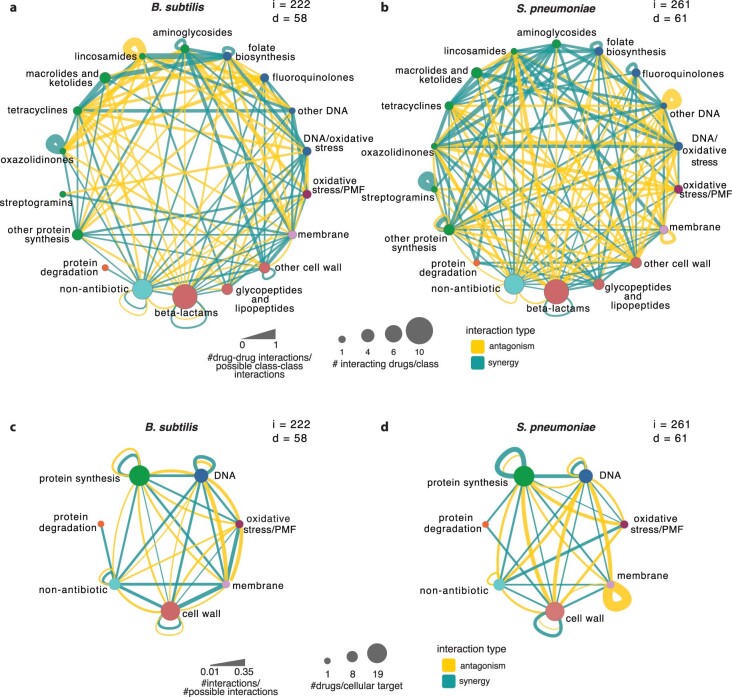
Drug interaction networks grouped according to drug class or targeted cellular processes for *B. subtilis* (a, c) and *S. pneumoniae* (b, d). Node size and colour and edge thickness are depicted as in Fig. 2a,b. i, number of interactions; d, drugs involved in the interactions (may differ from number of drugs tested in screen). Source data

**Extended Data Fig. 7:**
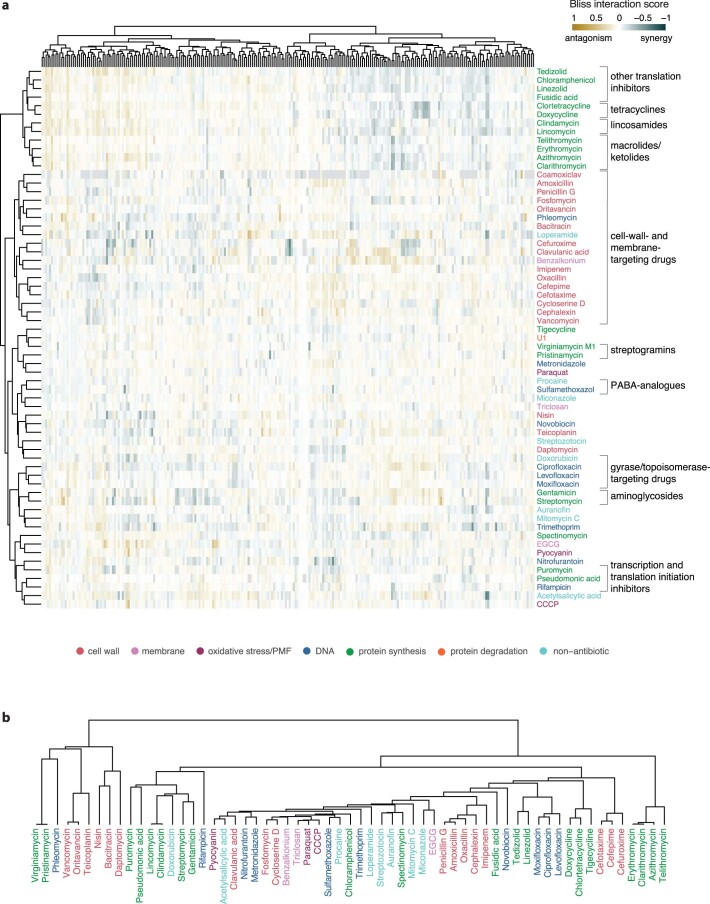
Drug interaction fingerprint recapitulates their functional and chemical classes. **a**, Drugs clustered according to their interactions with all other drugs in main screen. For each drug, quartile-based Bliss interaction scores (Methods) with all the other drugs (n = 65) in all four strains (x-axis) are considered; drug interaction fingerprints are then clustered according to their cosine similarity. All interaction cumulative scores are considered, regardless of their significance. Clusters enriched in drugs belonging to the same classes, targeting the same processes, and/or chemically similar, are highlighted. Negative, positive and neutral Bliss scores are depicted in shades of green, yellow, and in white, respectively. Combinations that were not tested in a given strain are in grey. **b**, Drug clustering according to their chemical structure similarity (Methods). In both panels, drugs are coloured according to their targeted cellular process (colour code as in Fig. 1). Source data

We built separate interaction networks for each of the three species tested and grouped drugs according to their class or cellular target (Fig. 2a,b and Extended Data Fig. 6). Although individual drug–drug interactions were rarely conserved (Fig. 1c), interactions between drug classes or targeted processes were more coherent in all three species. This functional concordance became even clearer when comparing drugs on the basis of all their interactions with other drugs. Interaction-based clustering better recapitulated drug functional classes (Extended Data Fig. 7a and Methods) than clustering on the basis of chemical structures (Extended Data Fig. 7b and Methods), suggesting that drug interactions capture more information on drug mode of action than their chemical features.

Since *S. aureus* is the most relevant Gram-positive species with respect to AMR-attributable deaths^2^, we systematically screened literature for reported drug interactions in this species. Out of 331 unique interactions detected across the two *S. aureus* strains in our study, we could find only 31 that have been previously reported, to the best of our knowledge (Fig. 2c and Supplementary Table 7). Some 55 further interactions have been reported in other bacterial species (Supplementary Table 7). Even when excluding those, our dataset revealed 127 novel synergies for *S. aureus* (and 118 antagonisms), a third of which (*n* = 39) was conserved in both strains. This confirms that the combinatorial space is a largely unexplored reservoir for improving antimicrobial efficacy.

Known interactions include many conserved synergies between drugs with the same targets (Fig. 1d), such as synergies between DNA biosynthesis inhibitors, protein synthesis inhibitors and cell-wall-targeting antibiotics (Fig. 2a,b). Among these latter, we confirmed the strong and previously reported synergy between two widely used antibiotics for *S. aureus*, cefepime and teicoplanin^27,28^, and validated it against several MRSA (methicillin-resistant *S. aureus*) clinical isolates belonging to worldwide prevalent clonal complexes and different infection sources (Supplementary Table 1), including a strain resistant to the last-resort antibiotic tigecycline (Fig. 2d and Supplementary Information). When we infected larvae of the greater wax moth *Galleria mellonella* with this MRSA strain, the combination protected the animals from succumbing to the infection in contrast to single drug treatments (Fig. 2e), confirming that the synergy works also in vivo.

Synergies between cell-wall-targeting drugs and translation inhibitors are cornerstones of anti-infective therapy against Gram-positive bacteria^29–32^. We could recapitulate some of these synergies: for example, conserved synergies between bacitracin or oritavancin and aminoglycosides in *S. aureus*. In line with current literature and concerns on general effectiveness^33–36^, we could not detect synergies between beta-lactams and aminoglycosides in any of the three species tested. Despite the prevalent assumption that these combinations are highly effective, synergies occur only for specific species (or strains) and depend on dosage, infection site and specific antimicrobial agents^33–36^. In contrast, fosfomycin strongly synergized with a diverse range of protein synthesis inhibitors (Supplementary Table 7) and could present an underexploited therapeutic resource against *S. aureus* (see Discussion).

Among the 300 previously unknown interactions we detected, 19 out of 23 tested were further confirmed in the extended 8 × 8 checkerboard benchmarking assays (9 of which were in both *S. aureus* strains) (Supplementary Table 4). Interestingly, adjuvants, such as clavulanic acid, or antibiotics used in clinics only in fixed-concentration combinations (trimethoprim and sulfonamides), exhibited a number of previously unknown synergies with other drugs, unveiling a so-far unexplored space for new combinations. As an example, we validated the strong synergy of teicoplanin with trimethoprim against several MRSA clinical isolates in vitro (Fig. 2d and Supplementary Information) and in vivo in a *G. mellonella* infection model (Fig. 2e).

### Target-specific synergies in Gram-positive/-negative species

**Fig. 3:**
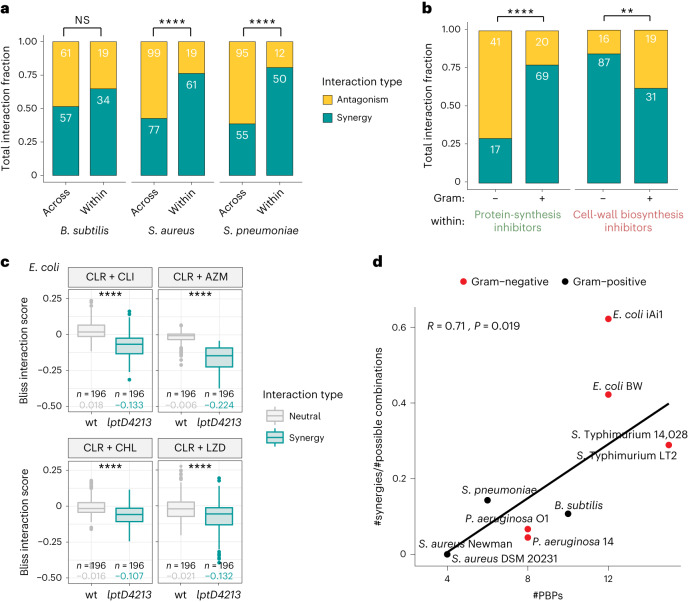
Synergies between drugs targeting the same cellular process. **a**, Drugs targeting the same biological process often interact synergistically, whereas antagonisms are prevalent between drugs targeting different processes (*S. aureus*: ****P = 9.9 × 10^−7^, *χ*^2^ test; *S. pneumoniae*: ****P = 4.7 × 10^−8^, *χ*^2^ test). Interaction numbers are indicated in white inside the bars. Non-antibiotic drugs (*n* = 8) are excluded from this analysis, as their targeted processes are heterogeneous or unknown. **b**, Gram-positive species exhibit frequent synergistic interactions between protein synthesis inhibitors, whereas cell-wall biosynthesis inhibitors predominantly synergize in Gram-negative species^11^. Prevalence of interactions between protein synthesis inhibitors and between cell-wall-biosynthesis inhibitors in Gram-negative and Gram-positive species is indicated as in Fig. 3a (for protein synthesis inhibitors: ****P = 1.8 × 10^−8^; for cell-wall biosynthesis inhibitors: **P = 0.0038, *χ*^2^ test). **c**, Protein synthesis inhibitors can also synergize in Gram-negative species when the drug permeability bottleneck is abolished. Synergistic combinations in Gram-positive species were tested in 8 × 8 broth microdilution checkerboards in wild-type *E. coli* and in the OM-defective *E.coli lptD4213* strain^40^. Interaction score distributions for each combination are significantly different between the two strains. Interactions were assigned with the same criteria used in the screen, with synergies corresponding to distributions with first quartile <−0.1. The first quartile value is shown in all cases. CLR, clarithromycin; CLI, clindamycin; AZM, azithromycin; LZD, linezolid; CHL, chloramphenicol (CLR + CLI: ****P = 2.2 × 10^−16^; CLR + AZM: ****P = 5.3 × 10^−13^; CLR + CHL: ****P = 2.2 × 10^−16^; CLR + LZD: ****P = 4.4 × 10^−8^, two-sided Wilcoxon test; box limits correspond to first and third quartiles, with the median marked, and whiskers extending to the most extreme data points up to 1.5 times the interquartile range (IQR). **d**, Differences in beta-lactam synergy prevalence between Gram-negative and Gram-positive species are related to differences in drug target redundancy, that is, the penicillin-binding proteins (PBPs) they encode in their genomes. Pearson correlation between number of PBPs and the frequency of synergies between beta-lactams for each strain tested (Supplementary Tables 8 and 9) and the *P* value of a two-sided permutation test (100,000 permutations) are shown. Correlations and *P* values when using one strain per species are shown in Supplementary Table 10. Source data

**Extended Data Fig. 8:**
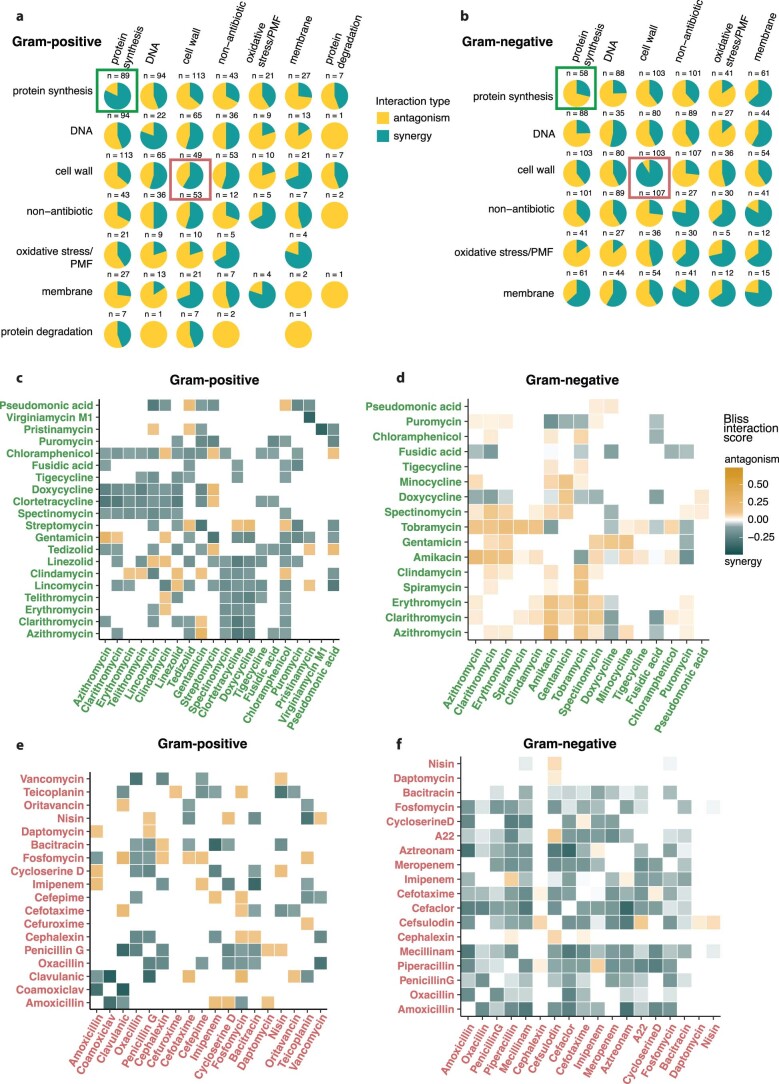
Interactions between drug functional classes in Gram-positive and Gram-negative species. **a**, Interactions between all drug classes (based on cellular target) in Gram-positive (**a**) and Gram-negative (**b**) species. The absolute count for each class-class interaction is indicated. PMF = proton-motive force. Interactions between drugs tested in all strains are considered. Interactions conserved across different strains are considered as distinct occurrences. **c-f**, Heatmaps of interactions between protein synthesis inhibitors (**c-d**) and between cell-wall biosynthesis inhibitors (**e-f**). Bliss interaction scores are averaged across strains if the same interaction is found in more than one strain. In rare cases in which opposite interactions are found in two different strains (n = 4, **c**; n = 2, **d**; n = 2, **e**; n = 8, **f**), the strongest one is shown here. Source data

Drugs belonging to the same class or targeting the same cellular process exhibited mainly synergistic interactions in all three species (Fig. 2a,b, and Extended Data Figs. 6 and 8a). Indeed, synergies between drugs targeting the same process were significantly enriched (Fig. 3a), in agreement with previous data on Gram-negative bacteria^11^. Targeting different facets of the same cellular process can bypass the inbuilt redundancy and robustness of biological processes^37^. Importantly, the targeted cellular processes that were more prone to synergies were distinct when comparing Gram-positive and Gram-negative species (Extended Data Fig. 8a,b). Synergies between protein synthesis inhibitors were specifically prevalent in Gram-positive species, whereas Gram-negative species were dominated by synergies between cell-wall inhibitors (Fig. 3b and Extended Data Fig. 8c–f). Since the drugs between the two screens largely overlapped and their targets are conserved in bacteria, we decided to further investigate the underlying reason for this difference.

Protein synthesis inhibitors are mostly used against Gram-positive bacteria, as they often cannot cross the outer membrane (OM) of Gram-negative bacteria. We reasoned that in Gram-positive species with no such permeability bottleneck, these drugs could synergize at their target level, the ribosome, as previously shown by combinations of genetic perturbations of translation^38^. By contrast, in Gram-negative bacteria, the OM permeability bottleneck probably masks such synergistic interactions and enriches for antagonisms, which are often due to a decrease in drug intracellular concentration(s)^11^. We confirmed this hypothesis by using the OM-defective *E.coli* mutant *lptD4213*, which is hyperpermeable to hydrophobic antibiotics and detergents^39,40^. Many of the interactions between macrolides and different classes of protein synthesis inhibitors became synergistic in this *E. coli* mutant background (Fig. 3c and Supplementary Information), demonstrating that drug uptake bottlenecks can change antibiotic interactions.

Interactions between beta-lactams were prominent in Gram-negative species, but rare in Gram-positive species (Extended Data Fig. 8e,f). Beta-lactams have different affinities to penicillin-binding proteins (PBPs)^41^. Interestingly, the number and type of PBP are largely different across bacterial species^41,42^, leading us to hypothesize that this redundancy (number of PBP paralogues) drives the observed difference. Indeed, the number of synergies in each strain tested correlated with the number of PBPs encoded in their genomes (Fig. 3d, and Supplementary Tables 8 and 9). The higher the number of PBPs, the higher the probability that combining beta-lactams with different affinities to the various PBPs will lead to a synergistic bypassing of the redundancy. Although this trend is based on a limited number of strains, it holds even when considering only one strain per species (Supplementary Table 10). While further studies are needed, we hypothesize that this target redundancy drives the synergies between beta-lactam antibiotics and that the difference we observed here between Gram-positive and -negative species probably depends on the number of PBPs in the species tested.

Altogether, these results support the known concept that drug interactions mirror key properties of cellular networks^43^, such as their functional modularity and redundancy, and reflect fundamental differences in cellular architecture across the Gram-positive/-negative divide.

### Interactions between non-antibiotics and antibiotics

**Fig. 4:**
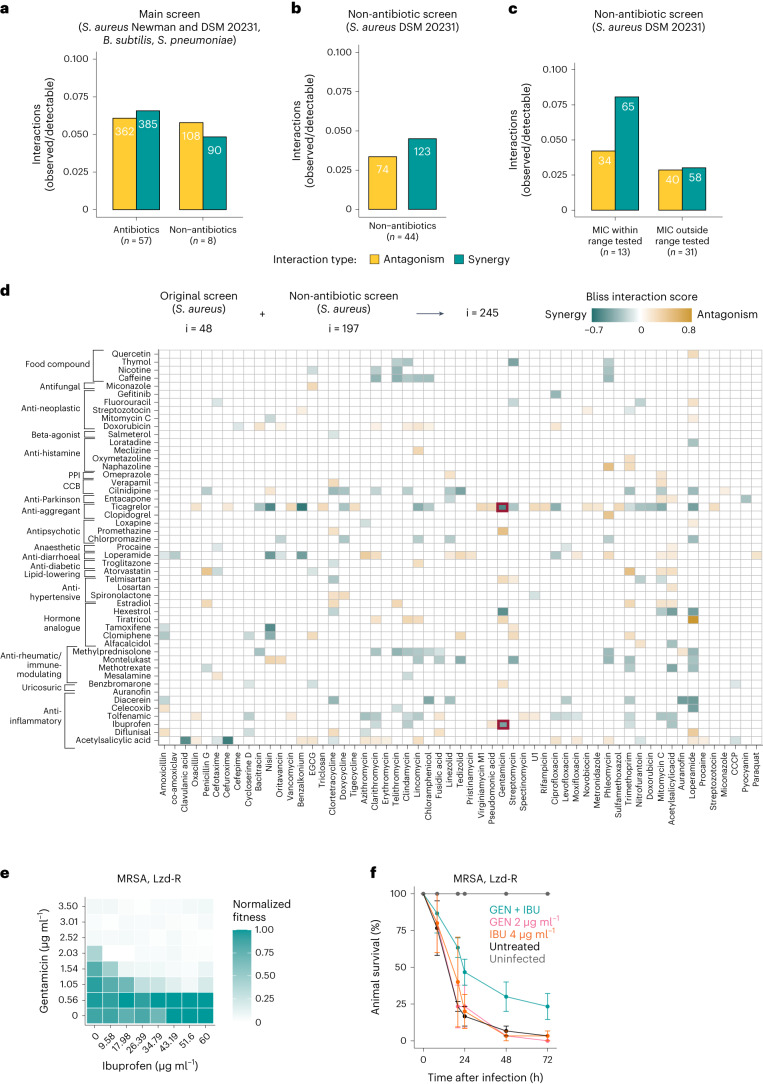
Interactions between non-antibiotic and antibiotic drugs in *S. aureus*. **a**, Interactions of non-antibiotic drugs between themselves and antibiotics are as common as interactions between two antibiotics. This motivated us to expand the non-antibiotic panel tested. Synergy and antagonism frequencies were calculated as in Fig. 1b. **b**, Interactions between non-antibiotics and antibiotics in the extended non-antibiotic screen in *S. aureus* DSM 20231. Additional non-antibiotic drugs (44) were screened in combination with 62 drugs belonging to the original drug panel, using the same experimental setup and the same data analysis pipeline, in *S. aureus* DSM 20231 (Methods). Synergy and antagonism frequencies were calculated as in Fig. 1b. **c**, Non-antibiotics with antibacterial activity, for which the MIC was among tested concentrations (*n* = 13), engage in more interactions than non-antibiotics for which there is no MIC or concentration was out of the tested range (*n* = 31). **d**, All interactions between non-antibiotics and antibiotics detected in *S. aureus* DSM 20231 in the original (i = 87) and in the extended (i = 197) non-antibiotic screen. PPI, proton pump inhibitors; CCB, calcium channel blockers; EGCG, epigallocatechin gallate. **e**,**f**, The non-steroidal anti-inflammatory ibuprofen (IBU) synergizes with gentamicin (GEN) in an MRSA clinical isolate with additional resistance to linezolid (Supplementary Table 1) in 8 × 8 broth microdilution checkerboards (**e**) and in the *G. mellonella* infection model (**f**). For checkerboards, the median fitness (OD_595_ at 7.5 h normalized by no-drug controls) across two biological replicates is shown (Supplementary Information). For *G. mellonella* experiments, results were obtained as in Fig. 2e. Data represent mean ± s.e. (*n* = 10 for each condition, three independent experiments). Source data

**Extended Data Fig. 9:**
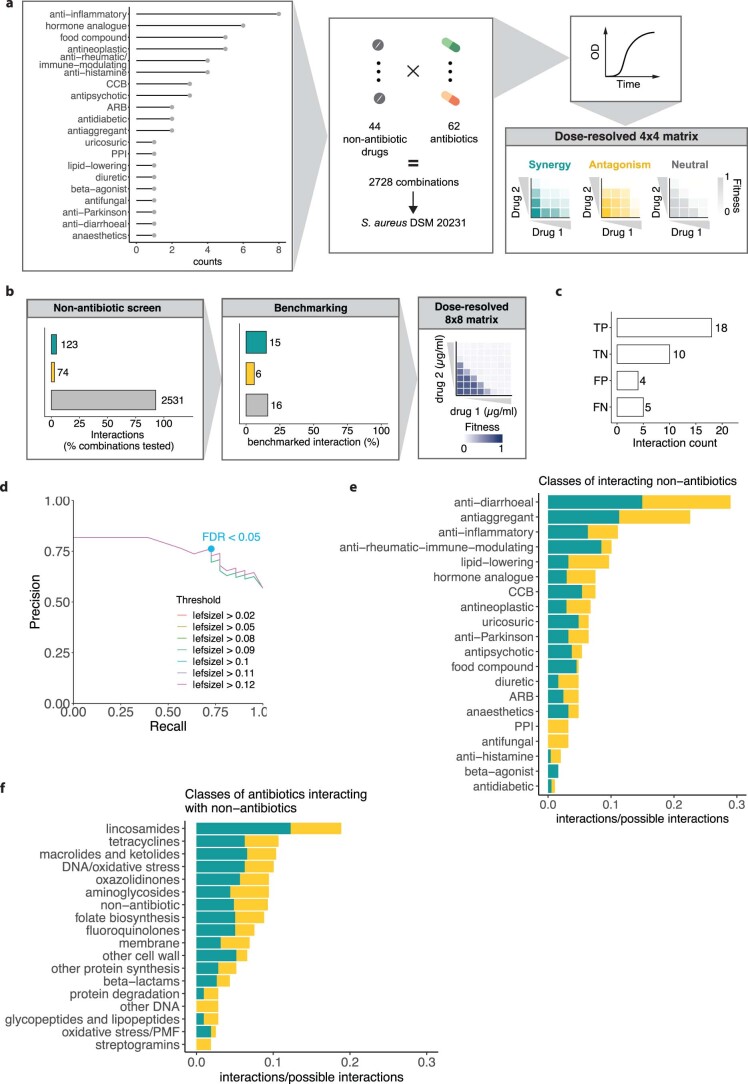
Non-antibiotic drug screen and benchmarking. **a**, Schematic representation of non-antibiotic drug high-throughput screen. 44 drugs belonging to different therapeutic classes (Supplementary Table 6) were tested in combination with 62 antibiotics at three concentrations in *S. aureus* DSM 20231. The resulting 2728 combinations were tested in broth microdilution (Methods). **b**, 37 drug combinations (Supplementary Table 6) were selected for benchmarking and tested in 8 x 8 concentration checkerboards (Methods). **c**, True-positive (TP), true-negative (TN), false-positive (FP) and false-negative (FN) abundance in the benchmarking set for optimal thresholds shown in **d**. **d**, Screen precision and recall against the benchmarking set are assessed for different effect-size thresholds. Precision-recall curves are shown for FDR intervals ranging from 0 to 1, increasing by 0.005. The chosen significance value for the screen (FDR < 0.05) is highlighted for the effect-size curve (|0.1|) providing best precision and recall. **e-f**, Interaction abundance for classes of non-antibiotics (**e**) and classes of antibiotics (**f**). Interactions detected in *S. aureus* DSM 20231 between antibiotics classes and 52 non-antibiotics tested in both the original screen (n = 8) and the extended screen (n = 44) were considered (n = 245). PMF, Proton-motive force; CCB, calcium-channel blocker; PPI, proton-pump inhibitors; ARB, angiotensin-receptor blocker. Synergies are depicted in green, antagonisms in yellow. Source data

Our drug interaction screen included eight non-antibiotic drugs, which exhibited a similar interaction frequency (11%) as antibiotics (13%) (Fig. 4a). This motivated us to expand the panel of non-antibiotic drugs tested and to explore the range of synergies and antagonisms antibiotics exhibit with commonly used non-antibiotic medications in *S. aureus*. We selected 44 drugs to include pharmaceuticals that can be co-administered with antibiotics in *S. aureus* infections or non-antibiotics with previously reported antibacterial activity against *S. aureus* (Supplementary Table 11). Altogether, we covered 19 therapeutic classes (Extended Data Fig. 9a and Supplementary Table 11), testing each drug in a range of three concentrations and against the panel of 62 drugs of the initial screen (2,728 drug–drug interactions, 4 × 4 dose matrix) in *S. aureus* DSM 20231. Concentrations were selected to fall within therapeutic plasma concentrations^44^, except for drugs with possible topical use, where higher concentrations were used. Interactions were scored and benchmarked as in the main screen (Methods, Supplementary Tables 12 and 13, and Extended Data Fig. 9a–d).

We confidently detected 197 interactions in this extended screen (Fig. 4b and Supplementary Table 12), an interaction frequency that was lower (7.8%) than that of the initial screen or the set of eight non-antibiotic drugs included therein (Fig. 4a). Since all eight non-antibiotic drugs included in the main screen were selected because they had reported antibacterial activity, we reasoned that this could account for their higher interaction rate. Indeed, for those drugs that had antibacterial activity on their own, the interaction frequency was double (12% vs 5.9%) (Fig. 4c). For all non-antibiotics tested in this work (*n* = 52), we detected 140 synergies and 105 antagonisms mainly with antibiotics (Fig. 4d). A small number of interactions (22 synergies and 23 antagonisms) were found between two non-antibiotics. Synergies offer a so-far unexploited potential for drug repurposing, whereas antagonisms expose risks of decreasing the efficacy of antimicrobial treatments.

The therapeutic classes that exhibited the highest number of interactions were anti-inflammatory drugs (*n* = 7, 4 of which were nonsteroidal anti-inflammatory drugs, or NSAIDs) and hormone analogues (*n* = 6) (Fig. 4d, Extended Data Fig. 9e and Supplementary Table 12), whereas for antibiotics, protein synthesis inhibitors dominated the interactions (Fig. 4d, Extended Data Fig. 9f and Supplementary Table 12). Interestingly, selective oestrogen-receptor modulators, such as the two triphenylethylene compounds tamoxifene and clomifene, shared their synergies with cell-wall-acting drugs and their antagonism with streptomycin. Hormone analogues engaged in several synergies (*n* = 11) and antagonisms (*n* = 17), suggesting an understudied impact that such commonly used drugs and potentially their natural counterparts, may have on the efficacy of antibacterial therapies^14,45^. For the anti-inflammatory drugs, only four interactions with acetylsalicylic acid were previously known: its synergy with cefuroxime^46^ and its antagonisms with ciprofloxacin, oxacillin and azithromycin^47–49^. We validated the synergy between ibuprofen and gentamicin also against MRSA clinical isolates, including a strain resistant to linezolid (a last-resort antibiotic for MRSA), in vitro and in vivo in a *G. mellonella* infection model (Fig. 4e,f and Supplementary Information).

### Ticagrelor has multiple effects on *S. aureus* physiology

Ticagrelor, a purine analogue anti-aggregant acting on the adenosine P2Y_12_ receptor^50^, had the highest number of interactions (*n* = 27) among the 44 non-antibiotics tested (Fig. 4d). Ticagrelor has been shown to improve clinical outcomes in patients with pneumonia and sepsis caused by Gram-positive bacteria^51,52^. Supported by different degrees of evidence, this effect can be because ticagrelor activates platelets upon systemic infection^52^, protects them from *S. aureus* toxin-mediated damage^53^, modulates their antibacterial properties^54^ and has direct bactericidal activity on *S. aureus* at high concentrations^55^. However, the mode of action of ticagrelor on *S. aureus* and its interactions with other drugs are largely uncharacterized.

**Fig. 5:**
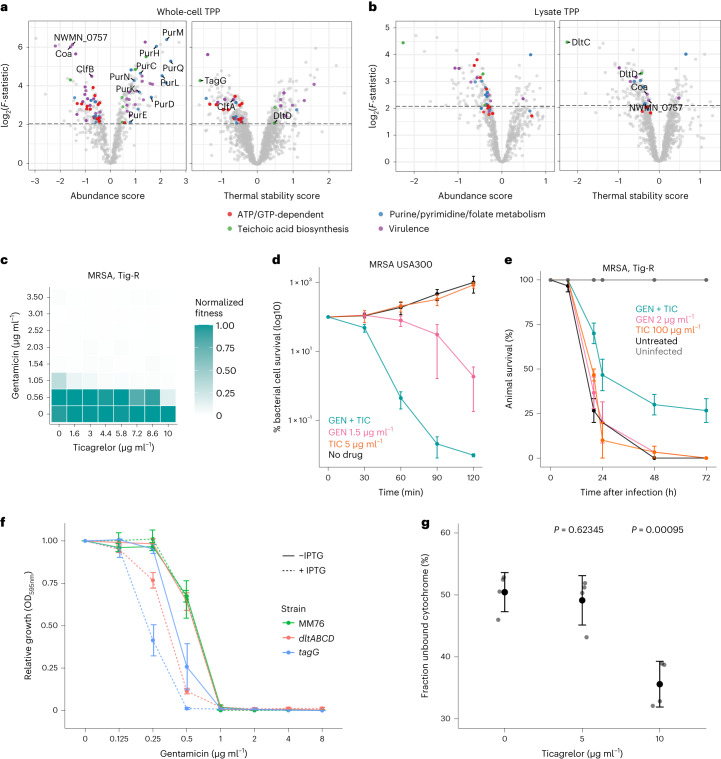
Ticagrelor alters *S. aureus* surface charge and potentiates cationic antibiotics. **a**,**b**, Volcano plots highlighting abundance or stability hits in whole-cell (**a**) and lysate 2D-TPP (**b**) data. The *x* axis represents the effect size of protein abundance or stability change^72^ (Methods) and the *y* axis corresponds to the statistical significance (log_2_(*F*-statistic)). For visualization purposes, when the *F*-statistic was 0, it was transformed to 1. **c**–**e**, Ticagrelor synergizes with gentamicin in vitro at growth inhibition (**c**, median fitness across two biological replicates, results obtained as in Fig. 2d, Supplementary Information), at killing level (**d**, mean ± s.e. of four biological replicates; drugs tested in combination at same concentration indicated for single drug treatments) and in vivo (**e**) against an MRSA isolate resistant to tigecycline (Supplementary Table 1). For *G. mellonella* experiments, results were obtained as in Fig. 2e. Data represent mean ± s.e. (*n* = 10 for each condition, three independent experiments). **f**, Growth (endpoint OD_595nm_, corresponding to the beginning of stationary phase for the control strain MM76, Methods and Supplementary Information) measured in the presence of serial 2-fold dilutions of gentamicin normalized by no-drug controls in the *S. aureus* IPTG-inducible knockdown mutants *dltABCD* and *tagG* and their control strain MM76 (Methods and Supplementary Table 1) in the presence or absence of 500 µM IPTG to induce maximal knockdown of the gene targeted (mean ± s.e. across four biological replicates). All strains were grown in the presence of 5 µg ml^−1^ erythromycin and 10 µg ml^−1^ chloramphenicol to maintain the CRISPRi plasmids^73^ (Methods). For all controls and full growth curves see Supplementary Information. **g**, *S. aureus* Newman surface charge changes upon exposure to ticagrelor. The fraction of positively charged unbound cytochrome *c* was measured after incubation of drug-treated and untreated samples (Methods); *n* = 4, mean ± s.e.; a two-sided Welch’s *t*-test was used to determine significance using the untreated samples as reference group. For all controls and cytochrome *c* standard curve, see Extended Data Fig. 10h. Source data

**Extended Data Fig. 10:**
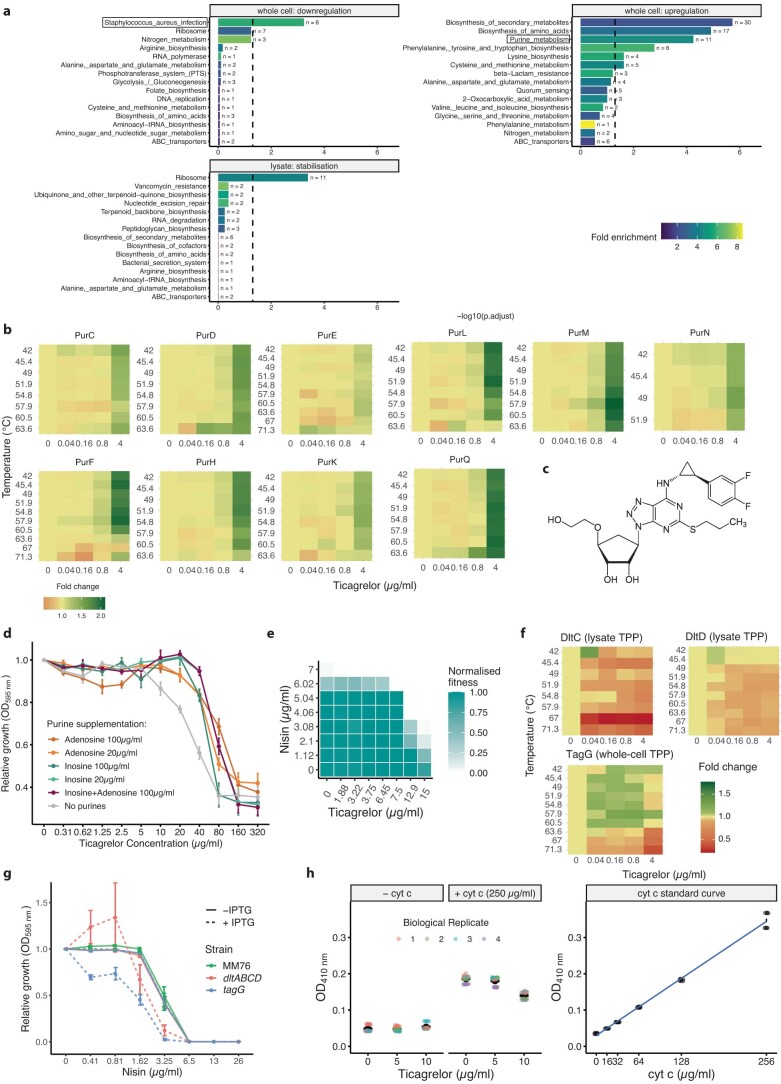
Ticagrelor affects purine and teichoic acid biosynthesis. **a**, KEGG enrichment of hits at 5% FDR from whole-cell or lysate samples. Only sets yielding significant enrichments (whole-cell down- and up-regulation, and lysate stabilization) are shown (Supplementary Table 7). The first 15 terms in order of significance are shown. The dashed lines mark the enrichment significance cut-off (adjusted p-value < 0.05, one-sided Fisher’s exact test). The number of protein hits is annotated for each term. **b**, Thermal stability profiles of members of the purine biosynthesis pathway. Protein fold change is shown for each temperature and ticagrelor concentration. **c**, Chemical structure of ticagrelor. **d**, Growth (endpoint OD_595nm_ after 11 h, corresponding to the beginning of stationary phase for the untreated control, Methods) measured in the presence of serial two-fold dilutions of ticagrelor in presence or absence of purines at the indicated concentration, normalised by no-drug controls, in *S. aureus* Newman in SSM9PR-defined medium (mean across four biological replicates; error bars represent standard error; Methods). **e**, Ticagrelor synergizes with nisin *in vitro* (median fitness across two biological replicates, results obtained as in Fig. 2d, Supplementary File). **f**, Thermal stability profiles of proteins involved in teichoic acid biosynthesis, represented as in **b**. **g**, Growth (endpoint OD_595nm_, corresponding to the beginning of stationary phase for the control strain MM76, Methods, Supplementary File) measured in the presence of serial two-fold dilutions of nisin, normalised by no-drug controls, in the *S. aureus* IPTG-inducible knockdown mutants *dltABCD* and *tagG* and their control strain MM76 (Methods), in presence or absence of 500 µM IPTG to induce maximal knockdown of the gene targeted (mean across four biological replicates; error bars represent standard error). All strains are grown in presence of 5 µg/ml erythromycin and 10 µg/ml chloramphenicol to maintain the CRISPRi plasmids^76^ (Methods). For all controls and full growth curves see Supplementary File. **h**, Raw data (OD_410nm_) from Fig. 5g are shown alongside all controls (samples not incubated with 250 µg/ml cytochrome c, cytochrome c standard curve including buffer control). The linear fit for the cytochrome c standard curve used to infer the unbound cytochrome C fraction in supernatants is shown (n = 4, mean and standard error of the mean are shown. Data points represent reads (n = 4 biological replicates for each condition, Methods).

To gain insights into the mode of action of ticagrelor and its interactions with other drugs, we used two-dimensional thermal proteome profiling (2D-TPP)^56–58^ in both lysate and whole-cell samples to investigate the direct and indirect effects of the drug, respectively (Methods). We observed a destablization of several ATP- and GTP-binding enzymes and transporters in both the whole cell and the lysate (Fig. 5a,b, Extended Data Fig. 10a, and Supplementary Tables 14 and 15) and the induction of many purine biosynthesis enzymes (PurC, PurD, PurE, PurF, PurH, PurK, PurL, PurM, PurN, PurQ) in live cells (Fig. 5a and Extended Data Fig. 10a,b). This is in agreement with ticagrelor being a purine analogue (Extended Data Fig. 10c). Furthermore, the MIC of ticagrelor increased upon supplementation of defined media with adenosine, inosine or their combination (Extended Data Fig. 10d), confirming that ticagrelor indeed interferes with purine metabolism in *S. aureus*.

The clinically observed effects of ticagrelor during *S. aureus* infection have not been linked so far to a direct effect of ticagrelor on *S. aureus* virulence. We discovered a pervasive impact of ticagrelor on *S. aureus* virulence determinants and regulators, many of which were downregulated and others destabilized (Fig. 5a, Extended Data Fig. 10a, and Supplementary Tables 14 and 15). In particular, we observed destablization in lysate and downregulation in whole-cell samples of key clotting factors secreted by *S. aureus* (ClfA, ClfB), the coagulase Coa and the von Willebrand-factor binding protein (vWBP) NWMN_0757 (ref. ^59^). These effects, evident at a clinically relevant ticagrelor concentration^55^, offer an alternative explanation for the beneficial effect of anti-aggregant therapy as an adjuvant in *S. aureus* systemic infection.

Ticagrelor exhibited a number of synergies and antagonisms with antibiotics in MSSA (methicillin-sensitive *S. aureus*; Fig. 4d). Interestingly, it broadly sensitized MSSA and MRSA to both cationic peptides (nisin; Extended Data Fig. 10e) and antibiotics (aminoglycosides, such as gentamicin; Fig. 5c and Supplementary Information). This potentiation effect of aminoglycosides occurred at low ticagrelor concentrations and was also evident at the killing level (Fig. 5d) and in vivo, during infection of *G. mellonella* (Fig. 5e). Since aminoglycosides need energy to cross the bacterial membrane^60^, we wondered whether ticagrelor acted at that level, for example, by modulating the cell surface charge and increasing aminoglycoside uptake. Consistent with this hypothesis, two proteins involved in the lipoteichoic acid (LTA) d-alanylation^61^, DltC and DltD, were destabilized in the TPP lysate data, and TagG, a subunit of the cell wall teichoic acid (WTA) translocase, was destabilized in the whole-cell sample (Fig. 5a,b and Extended Data Fig. 10f). Disruption of teichoic acids and specifically, inactivation of *dltA*, *dltB* and *dltC* have been shown to sensitize *S. aureus* to cationic compounds because of an increase in the net negative charge of *S. aureus* surface^62–64^. We detected a decrease in the MIC of the aminoglycoside gentamicin and the cationic antibiotic nisin in isopropyl β-D-1-thiogalactopyranoside (IPTG)-inducible CRISPRi knockdown mutants of both the *dltABCD* operon and *tagG* (Methods, Fig. 5f and Extended Data Fig. 10g). Ticagrelor treatment also increased the binding of positively charged cytochrome *c* to intact *S. aureus* cells (Fig. 5g and Extended Data Fig. 10h). Thus, ticagrelor treatment impacts the thermal stability and presumably the activity of proteins involved in WTA flipping and LTA d-alanylation, leading to an increase in the surface net negative charge of *S. aureus*. This leads to potentiation of the uptake of cationic antibiotics, such as aminoglycosides and nisin.

## Discussion

In this study, we systematically profiled drug combinations against three Gram-positive bacterial species. We tested multiple compounds from each of the main antibiotic classes used to treat infections caused by Gram-positive pathogens, as well as neglected antibiotics, commonly used antibiotic adjuvants and promising non-antibiotic drugs with reported antibacterial activity. Combinations were tested in a dose-dependent manner and interactions were assessed in a quantitative manner. We report a plethora of drug–drug interactions, most of which have not been reported before. Some identified synergies were also effective against multiple MDR clinical isolates that we tested and during infections in vivo. It should be noted that this does not mean that such synergies will be effective against all MDR isolates (as we show here and have shown before^11^, interactions can be strain-specific) and combinations should be tested on the particular clinical isolate they are targeted to before their use, as done in the clinic for single drugs.

The dataset generated here can form the basis for future experiments to mechanistically dissect key interactions or assess potential for clinical application. For example, some of the synergies and antagonisms identified may guide future broad-spectrum empiric treatments, when antibiotic regimens are started without knowledge of the pathogen in time-sensitive contexts (for example, sepsis). Fosfomycin synergies that are strong and conserved across the Gram-positive/-negative divide are good candidates, as they are more likely to be broadly effective. Fosfomycin is increasingly used in clinics^65^, but rarely in combinations. To enable further use of this resource, we made it browsable in a user-friendly interface.

We compared results with those we obtained previously using a similar screen with three Gram-negative species^11^. The confidence and depth level of these comparisons are high, since the two studies have similar experimental and data analysis design, including the drugs tested. As for Gram-negative species, we found that drug interactions were largely species-specific for Gram-positive species, with synergies tending to be more conserved than antagonisms and driven by antibiotics sharing general cellular targets. In contrast, antagonisms were more common between antibiotics of different cellular targets and are less conserved, presumably because they are driven by interactions at the level of drug concentration^11^. Overall, only a small number of interactions is conserved across Gram-positive and -negative species. Such interactions are more likely to be driven by interactions at the drug target and may thus hold true for strains and species not tested here, providing a high-confidence set for future exploitation. Differences in cell surface organization (for example, the outer membrane posing a permeability barrier for hydrophobic compounds) or in the degree of redundancy in cell-wall-building enzymes can explain some of the strong synergies observed specifically in Gram-positive or Gram-negative species. Our ability to replicate Gram-positive-specific synergies in hyperpermeable Gram-negative bacteria, together with previous evidence on the strong dependence of antagonisms on drug permeability^11^, suggests that synergies rely more on drug targets and antagonisms more on drug intracellular concentrations. The exact degree to which this is true and whether this makes antagonisms less conserved (drug cellular targets are more conserved than their transport mechanisms) remain to be systematically assessed. In any case, this is presumably the reason why antagonisms were more frequent than synergies in Gram-negative species^11^ but not here: synergies based on drug target are easier to detect in Gram-positive bacteria, and antagonisms are less common, as there are fewer permeability bottlenecks to overcome.

We also assessed combinations of approved non-antibiotic drugs with antibiotics in a dose-dependent manner in *S. aureus*. Although the interaction potential was lower for drugs without antibacterial activity, the vast majority of synergies that we detected were previously unknown. Although non-antibiotic drugs have been proposed as anti-infective adjuvants for decades^4,5^, their in vivo efficacy and molecular basis of action are only known for a few examples^5,7,18,55,66,67^. We focused on the anti-aggregant ticagrelor, whose repurposing as an anti-infective adjuvant for Gram-positive bacteria has been recently proposed^53,68^. While the in vivo benefit of ticagrelor for systemic infections has been documented^51,55^, we identified 13 additional synergies with antibiotics in *S. aureus*, and provided molecular insights into how ticagrelor affects *S. aureus* physiology and potentiates positively charged antibiotics, such as aminoglycosides or nisin.

Drugs are regularly combined to treat patients in the clinic, not only in rationally designed therapeutic schemes, but also extemporarily in poly-treated patients^14^. Although known pharmacokinetic interactions that have been documented in humans are routinely avoided, it is assumed that interactions between drugs in bacteria will not impact anti-infective efficacy. We detected both synergies and antagonisms between commonly administered non-antibiotic drugs and antibiotics against *S. aureus*. These antagonisms could potentially decrease the efficacy of antibiotic therapies and increase the probability of emergence of resistance. Overall, it is important that drug interactions are investigated not only at the level of growth inhibition, but also at the levels of killing and clearing of an infection, as the interaction outcome might differ^10^.

It has recently been proposed that the attenuation of antibiotic efficacy (antagonism) could be used to reduce the collateral damage of antibiotics on commensal bacteria^69^. In our screen, loperamide had the most interactions with antibiotics. Although its potential use as an adjuvant for specific antibiotics and its antibacterial mode-of-action are known^5^, we detected an additional broad antagonism with macrolides. Loperamide and macrolides are often co-administered for travellers’ diarrhoea^66^, which is caused by Gram-negative enteric pathogens. It is tempting to speculate that part of the beneficial effect of this combination results from the protection of Gram-positive commensal gut species from macrolide action.

In summary, we present a systematic and quantitative account of drug interactions against important Gram-positive species. Thereby, we discovered a number of potent synergistic combinations that are effective against clinical MDR isolates. We also investigated mechanisms of selected interactions. In an era where novel antibiotic development faces technical and economic hurdles, and new antimicrobial strategies are urgently needed, we propose that systematic drug interaction profiling might offer alternative solutions to treat bacterial infections. Extending the systematic testing of drug (antibiotic or non-antibiotic) interactions to additional bacterial species will improve our understanding of drug interaction conservation and mechanisms, and inform tailored treatments for bacterial infections.

## Methods

### Strains and growth conditions

All strains used in this study are listed in Supplementary Table 1. *B. subtilis* subsp. *subtilis* 168 (ref. ^70^) was kindly provided by C. A. Gross, all MRSA clinical isolates by S. Göttig, *S. pneumoniae* D39V^71^ by J.-W. Veening and *S. aureus* USA300 by D. Lopez. *Staphylococcus aureus* subsp. *aureus* Newman^72^ was purchased from NCTC (NCTC 8178) and DSM 20231 (ref. ^73^) (ATCC 12600 ^T^, NCTC 8532) from DSMZ.

For all experiments and unless otherwise specified, *S. aureus* strains were grown in tryptic soy broth (TSB, 22092, Merck-Millipore), *B. subtilis* in LB Lennox and *S. pneumoniae* in CY medium, as adapted from ref. ^74^. All species were grown at 37 °C with vigorous shaking (850 r.p.m.), except for *S. pneumoniae*, which was grown without shaking. The ticagrelor purine supplementation experiments in *S. aureus* Newman were conducted in SSM9PR-defined medium supplemented with 1% glucose^75^.

### Inducible knockdown strain construction

A two-plasmid CRISPR interference system was used to knock down gene expression of selected genes in *S. aureus* Newman^76^. In these strains, *dcas* was expressed from an IPTG-inducible promoter on plasmid pLOW, while single guide RNAs (sgRNAs) were expressed from a constituted promoter on a plasmid derived from pCG248. The sgRNA target sequences were TGTCTAACAGCAATGCTTTG for *dltABCD* and AAACCATAATTTGCATAACA for *tagG*, and ATAGAGGATAGAATGGCGCC for the non-target control MM76 (Supplementary Table 1).

### MIC and IC_50_ determination

MICs and IC_50s_ were tested in all strains for the main screen (Supplementary Tables 1 and 2). Drugs were 2-fold serially diluted in 11 concentrations, and 32 no-drug control wells were included in each plate. Experiments were conducted in flat, clear-bottom 384-well plates (781271, Greiner BioOne), with a total volume of 30 µl for *S. aureus* and *B. subtilis* and 55 µl for *S. pneumoniae*. Volumes were optimized for each strain to achieve good dynamic range for growth and minimize risk of cross-contamination between wells. Plates were inoculated with a starting OD_595_ of 0.01 from an overnight culture. All liquid handling was performed using a Biomek FX liquid handler (Beckman Coulter). Plates were sealed with breathable membranes and incubated at 37 °C. OD_595_ was measured every 30 min for 14 h using a Filtermax F5 multimode plate reader (Molecular Devices). OD_595_ values were background subtracted (using the OD_595_ value at the first timepoint). The timepoint corresponding to entry into stationary phase in no-drug control wells was selected for each strain: 8 h for both *S. aureus* strains, 5.5 h for *B. subtilis* and 3.7 h for *S. pneumoniae*. For each drug concentration and strain, the OD_595_ value at this timepoint was then divided by the robust mean^77^ of the corresponding values of the no-drug controls for each strain. For each drug, IC_50s_ were then calculated after fitting a four-parameter log-logistic model using the R package drm^78^. The MIC was considered as the lowest concentration at which growth was inhibited. Experiments were conducted in biological duplicates.

### High-throughput screen of drug combinations

Sixty-two drugs, hereafter designated as recipients, were arrayed in flat, clear-bottom 384-well plates in three 2-fold serial dilutions and 2 technical replicates (up to 2 recipient drugs were removed from the data of the different strains for quality control reasons). Concentrations were selected according to MICs, with the highest concentration corresponding to the MIC, and the intermediate and lowest concentration corresponding to half and a quarter of MIC, respectively (Supplementary Table 2). Plates were kept frozen and were defrosted upon each experimental run, when the same 62 drugs and in the same three concentrations were added as donor drugs (one drug at one concentration for each recipient plate). A few drugs were screened only as donors: the combinations co-amoxiclav and cotrimoxazole in *B. subtilis* and *S. aureus* DSM 20231; co-amoxiclav, clavulanic acid, pseudomonic acid and cefuroxime in *S. aureus* Newman. All donor drugs were tested in two biological replicates. Control wells were included in each plate (6 no-drug wells, 3 plain medium wells and 3 wells containing only the donor drug). After the addition of donor drugs, plates were inoculated with cells. Handling, inoculation, growth conditions, plate incubation and OD_595_ measurements were performed as in MIC determination.

For the adjuvant screen, 44 non-antibiotic drugs (Supplementary Table 11) were tested against the same 62 recipient drugs of the main screen for *S. aureus* DSM 20231. Antibiotics were tested at the same concentrations as in the main screen. Non-antibiotics concentrations were selected to fall within therapeutic plasma concentrations^44^ (Supplementary Table 11).

### Data analysis

Data analysis was adapted from ref. ^11^. Growth curves were processed as described in Extended Data Fig. 1: the background was subtracted from all OD_595_ measurements on a well-by-well basis using the first measurement obtained. Abnormal spikes in OD values of the first three timepoints occurred in *S. pneumoniae* in a small fraction of wells due to bubble formation in the medium or plate condensation. These early local peaks in OD curves were identified and replaced with the median of OD values (of corresponding timepoints) estimated from the wells not affected by such artefacts within the same plate. When more than one in the first four timepoints was affected, those wells were identified as non-monotonically increasing OD_595_ values across the first four timepoints, and their background was estimated as the median first-timepoint OD_595_ of artefact-free wells (monotonically increasing across the first four timepoints).

A single-timepoint OD_595_ value was selected at the transition between exponential and stationary phase as in the IC_50_ determination and used to derive fitness measurements that captured effects both on growth rate and maximum yield. OD-based endpoints correlated well with AUC-based fitness measurements (Pearson correlation 0.96) (Extended Data Fig. 2e), whereas fitness based on growth rate alone (calculated by fitting a Baranyi model^79^) correlated worse with either AUC- or endpoint OD-based measurements (Pearson correlation 0.68 and 0.75, respectively) (Extended Data Fig. 2f,g). We then verified which measurement between AUC and endpoint OD was most accurate as compared to the screen benchmarking and ultimately chose the latter, which led to higher precision and recall (Extended Data Fig. 2d).

This value was then divided, per plate, by the robust mean^77^ of the 6 no-drug controls (no-drug control hereafter), obtaining 3 fitness measures for each drug concentration pair: *f*_1_, fitness upon exposure to drug 1; *f*_2_, fitness upon exposure to drug 2; and *f*_1,2_, fitness in presence of drug 1 + drug 2. On the basis of these values, further quality control was again performed, correcting fitness increase artefacts (maximum fitness was set to 1) and removing plates with poor technical replicate correlation (Pearson correlation <0.7). *f*_1_, *f*_2_ and *f*_1,2_ were used to calculate interaction scores using the Bliss model^19^. The choice of this model over other available quantification methods was driven by the following considerations: (1) the three measurements obtained for drug dose responses are not sufficient for accurate quantification using alternative models (for example, the Loewe model^80^) and (2) the Bliss model can more accurately account for single drugs with no effect (such as most non-antibiotic drugs included in the screen).

Bliss (*ε*) scores were calculated as follows:

1 $$\varepsilon ={f}_{{\rm{d1}},{\rm{d2}}}-{f}_{{\rm{d1}}}\times{f}_{{\rm{d2}}}$$

where *f*_d1d2_ corresponds to the observed fitness in the presence of the drug combination, and *f*_d1_ and *f*_d2_ correspond to the fitness in the presence of drug 1 and drug 2, respectively.

Assuming that most drugs interact neutrally, single drug fitness for both donor and recipient drugs can also be inferred from combination fitness, by minimizing the sum of squared residuals of the Bliss independence model as follows:

2 $$\left\{\,{f}_{\rm{d}1},{f}_{\rm{d}2}\right\}={\rm{arg }}\min \sum _{\rm{d}1,\rm{d}2}{{{\Big\Vert}}{f}_{\rm{d1,d2}}-{f}_{\rm{d}1}\times{f}_{\rm{d}2}{{\Big\Vert}}}^{2}$$

Experimentally measured and estimated fitness values were very similar for donor (Extended Data Fig. 2h) and recipient (Extended Data Fig. 2i) drugs, and we used the estimated measures since those were more robust to noise; experimental controls were limited for donor drugs (3 single-drug control wells) and sometimes biased for recipient drugs, as a single problematic plate in the batch was sufficient to generate noise.

When no data were discarded upon quality control, the number of Bliss scores obtained for each combination was 72, composed of 3 × 3 (in the 2D concentration space) × 2 technical replicates × 2 biological replicates × 2 replicates with drugs tested as donor or recipient. Hit calling was performed using a resampling procedure with 10,000 repetitions for each combination tested, where the *ε* distribution for each combination was compared with the resampled Bliss scores using Wilcoxon rank-sum test in each iteration^11^. Hits correspond to combinations with False Discovery Rate (FDR) < 0.05.

As before^11^, we coupled this significance threshold to an effect-size threshold. For each combination, we defined a cumulative score using the quartiles of its distribution of *ε* scores. We tested the performance of different thresholds in precision and recall upon screen benchmarking, and identified |0.1| as the best threshold, with precision of 0.87 and recall of 0.68 (Extended Data Fig. 3c,d). Accordingly, synergies were assigned if the first quartile of the *ε* distribution was <−0.1 and antagonisms if the third quartile exceeded 0.1. We could increase the screen recall by leveraging the presence of two strains belonging to the same species in the case of *S. aureus*, as previously described for Gram-negative species^11^. We defined an additional set of hits (weak and conserved), meeting significance and effect-size thresholds in one strain, but with lower effect size in the other strain. A cut-off of |0.08| allowed us to maintain the same precision and increase the recall to 0.72.

Data analysis was implemented in R (v.4.1.2)^81^ and RStudio (v.2021.09.1)^82^, and networks were created with Cytoscape (v.3.8.2)^83^.

### Interaction detection calculation

Interaction detection rates were calculated by dividing the number of detected interactions by the number of combinations for which interactions could be observed according to the mapped fitness space^11^. Synergies could not be observed when the expected fitness of a drug combination (defined as the product of single-drug fitness values in equation (1)) was lower than 0.1, while antagonisms could not be detected for expected combination fitness higher than 0.9 (2.1 and 3.3% of the 7,986 combinations tested, respectively).

### Screen benchmarking and 8 × 8 checkerboard assays

Combinations were tested in the same experimental conditions as in the screen, but at higher concentration resolution. Drugs were diluted in 8 concentrations spanning linearly spaced gradients to assemble 8 × 8 checkerboards for each combination tested (the highest concentration used can be found in Supplementary Table 4). All experiments were conducted in at least 2 technical and 2 biological replicates. Data were analysed with the same pipeline as in the screen (Methods). For this concentration-resolved set, we also calculated interactions according to the Loewe additivity model using the BIGL R package^84^. Using fitness (relative growth) as a response variable, dose responses of monotherapies were fit with a four-parameter logistic regression, with upper and lower boundaries set at 1 and 0, respectively. The null model for the expected response surface was the alternative Loewe generalization^84^. The prediction covariance matrix was estimated using 200 bootstrap iterations, and the interaction sign and significance were assessed for each concentration combination using the average deviation from the null model as previously described^84^. The errors for drug combination effects were assumed to be normally distributed. For 24 combinations where one of the drugs had no effect, Loewe could not be applied. Combinations for which the Loewe model could be applied but was unreliable were removed from later analyses. The model was deemed unreliable when: (1) an interaction effect was estimated outside of its confidence interval (due to bias in the Shannon entropy estimate^85^) and (2) the majority of individual dose combinations deviating from the Loewe null hypothesis disagreed with the direction of an overall global deviation. A separate analysis was carried out to visualize Loewe isoboles. At each concentration of one of the drugs in combination, an individual four-parameter log-logistic model was fit to the dose response of the other drug. To visualize the lines of additive (according to Loewe’s model) and experimentally observed effects, for each combination, an appropriate magnitude of drug effect was chosen according to the relevant effect sizes (that is, using lower or higher fitness values for stronger or weaker drug effects, respectively) (Extended Data Fig. 4c,d and Supplementary Information).

### Drug clustering

Drug–drug interaction profiles were clustered according to the cosine similarity of quartile-based Bliss interaction scores of each drug pair in each strain. Scores from all interactions were considered, regardless of their statistical significance. For the clustering based on chemical structures, drugs were clustered according to their Tanimoto similarity^86^ using 1,024-bit ECFP4 fingerprints^87^.

### Phylogeny analysis

To calculate the percentage sequence identity between bacterial species, the genomes of *B. subtilis* 168, *S. aureus* Newman, *S. aureus* DSM 20231, *S. pneumoniae* D39, *E. coli* K-12, *S. enterica* serovar Typhimurium LT and *P. aeruginosa* PAO1 were downloaded from NCBI and 40 universal single-copy marker genes were extracted using the fetchMG script^88^. The 40 marker genes were selected from a previous publication for their ability to characterize prokaryotic species^24^, and they encode for ubiquitous functions similar to transfer RNA synthetases or are ribosomal proteins (EggNOG COGs: COG0012, COG0016, COG0018, COG0048, COG0049, COG0052, COG0080, COG0081, COG0085, COG0087, COG0088, COG0090, COG0091, COG0092, COG0093, COG0094, COG0096, COG0097, COG0098, COG0099, COG0100, COG0102, COG0103, COG0124, COG0172, COG0184, COG0185, COG0186, COG0197, COG0200, COG0201, COG0202, COG0215, COG0256, COG0495, COG0522, COG0525, COG0533, COG0541, COG0552). The concatenated sequences (all 6 genomes contained exactly 40 marker genes) were used to calculate percentage nucleotide sequence identity with vsearch^89^ and to create a phylogenetic tree. To this end, a multiple sequence alignment was created using MUSCLE (v.3.8.1551)^90^ with default parameters. Finally, a maximum-likelihood phylogenetic tree was constructed using the online tool PhymL (v.3.0)^91^ with default parameters. To evaluate interaction conservation, only the 46 drugs tested both in Gram-positive and Gram-negative species (Supplementary Table 2) were considered.

### Evaluation of drug combination therapy using the *G. mellonella* infection model

Larvae of the greater wax moth (*Galleria mellonella*) at their final instar larval stage were used for evaluation of selected drug combinations to assess their efficacy against MRSA in vivo. Larvae were purchased from UK Waxworms and Mucha Terra. Stock solutions of cefepime, gentamicin, ibuprofen, teicoplanin and trimethoprim were freshly prepared as described for the in vitro experiments (Supplementary Tables 2 and 11), except for ticagrelor which was dissolved in 50 mM ethanol and diluted in distilled water to the required concentration. We opted for moderately virulent MRSA isolates in the larva model to be able to detect both the therapeutic effects of the antibiotic and possible synergies or antagonisms. Drug toxicity was preliminarily assessed by injecting larvae with serial dilutions of single drugs and combinations. Concentrations at which no toxicity was observed (that is, ≥90% survival rate at 72 h post injection) were selected for further experiments. The MRSA strains were cultivated in brain heart infusion medium and collected at an OD_600_ of 0.5. Bacteria were washed twice with PBS and adjusted to an OD_600_, which corresponded to a lethal dose of ~75% (LD_75_) of the larvae after 24 h (~10^7^ colony-forming units, CFUs). Ten larvae per condition were injected with 10 µl of the bacterial cell suspension or PBS (referred to as uninfected control) into the haemocoel via the last left proleg using Hamilton precision syringes. After 1 h, 10 µl of single drug combinations or vehicle were injected into the last right proleg at the following drug concentrations: teicoplanin 1 µg ml^−1^, trimethoprim 250 µg ml^−1^, cefepime 0.025 µg ml^−1^, gentamicin 2 µg ml^−1^, ibuprofen 4 µg ml^−1^, ticagrelor 100 µg ml^−1^. The survival of *Galleria* larvae was monitored at the indicated timepoints by two observers independently. Each strain–drug combination was evaluated in three independent experiments.

### Time-kill experiments

Overnight cultures of *S. aureus* USA300 were diluted 1:100 in 20 ml of TSB medium, incubated for 1 h in flasks at 37 °C with continuous shaking and diluted again 1:100 in 20 ml prewarmed TSB with ticagrelor (5 µg ml^−1^), gentamicin (1.5 µg ml^−1^), their combination or without drugs. Of serial 10-fold dilutions of cultures, 50 µl were plated on TSA plates every 30 min for 2 h. Cell viability was determined by counting CFUs after plates were incubated overnight in four independent experiments.

### 2D-TPP

Bacterial cells were grown overnight at 37 °C in TSB and diluted 1,000‐fold into 50 ml of fresh medium. Cultures were grown at 37 °C with shaking until OD_578_ ~ 0.6. Ticagrelor at the desired concentrations (0.04, 0.16, 0.8 and 4 µg ml^−1^) or vehicle was added and cultures were incubated at 37 °C for 10 min. Cells were then pelleted at 4,000 × *g* for 5 min, washed with 10 ml PBS containing the drug at the appropriate concentrations, resuspended in the same buffer to an OD_578_ of 10 and aliquoted to a PCR plate. The plate was then exposed to a temperature gradient for 3 min in a PCR machine (Agilent SureCycler 8800), followed by 3 min at room temperature. Cells were lysed with lysis buffer (final concentration: 50 μg ml^−1^ lysostaphin, 0.8% NP‐40, 1x protease inhibitor (Roche), 250 U ml^−1^ benzonase and 1 mM MgCl_2_ in PBS) for 20 min with shaking at room temperature, followed by five freeze–thaw cycles. Protein aggregates were then removed by centrifuging the plate at 2,000 × *g* and filtering the supernatant at 500 × *g* through a 0.45 µm filter plate for 5 min at 4 °C. Protein digestion, peptide labelling and MS-based proteomics were performed as previously described^57^.

### 2D-TPP data analysis

Data were pre-processed and normalized as previously described^56^. Raw MS files were processed using isobarQuant^92^. Peptide and protein identification were performed using Mascot 2.4 (Matrix Science) against the *S. aureus* Newman strain Uniprot FASTA (Proteome ID: UP000006386), modified to include known contaminants and the reversed protein sequences. Data analysis was performed in R using the package TPP2D^93^ as previously described^94^. Briefly, to identify stability changes, a null model allowing the soluble protein fraction to depend only on temperature, and an alternative model corresponding to a sigmoidal dose-response function for each temperature step were fitted to the data. For each protein, the residual sums of squares (RSS) of the two models were compared to obtain an *F*-statistic. FDR control was performed with a bootstrap procedure as previously described^94^. The abundance or thermal stability effect size was calculated for each protein as follows:

3 $${\rm{sign}}\left(\kappa \right)\bullet \sqrt{{{\rm{RSS}}}^{0}-{{\rm{RSS}}}^{1}}$$

where *κ* is the slope of the dose-response model fitted across temperatures and drug concentrations, and RSS^0^ and RSS^1^ correspond to the residual sums of squares of the null (pEC50, i.e., the negative logarithm of the EC50, linearly scaling with temperature) and alternative models, respectively^95^.

### Kyoto Encyclopedia of Genes and Genomes (KEGG) enrichment

The *S. aureus* Newman proteome was annotated using KEGG^96^ (release 100.0, 1 October 2021). Proteins with missing KEGG annotation were preliminarily removed. A one-sided Fisher’s exact test was then used to test the enrichment of input protein sets (hits corresponding to FDR < 0.05) against the background (all detected proteins) for each term. The *P* values were corrected for multiple testing using the Benjamini–Hochberg procedure. The analysis was performed in R using the packages KEGGREST^97^, EnrichmentBrowser^98^ and clusterProfiler^99^.

### Ticagrelor MIC upon purine depletion and supplementation

Ticagrelor (SML2482, Sigma-Aldrich) MIC was measured upon purine supplementation in *S. aureus* Newman as described above in SSM9PR-defined medium supplemented with 1% glucose^75^ in flat, clear-bottom 384-well plates with a final volume of 30 µl. Adenine and inosine were added at 20 and 100 µg ml^−1^, respectively, or in combination, both at 100 µg ml^−1^. Experiments were conducted in four biological replicates. A single-timepoint OD_595_ at the transition between exponential and stationary phase (13.5 h) was used to derive dose-response curves after normalization to the respective no-drug control for each condition.

### Gentamicin and nisin MIC measurements in *dltABCD* and *tag*G knockdown mutants

For gentamicin and nisin MIC measurements, *dltABCD* and *tagG* IPTG-inducible knockdown mutants (Methods and Supplementary Table 1) were grown in 2-fold dilutions of nisin and gentamicin in the presence of erythromycin (5 µg ml^−1^) and chloramphenicol (10 µg ml^−1^) for plasmid maintenance. IPTG (500 µM) was used to achieve maximal dCas9 expression and thereby, knockdown of the targeted gene. The parent *S. aureus* Newman and the control strain MM76 (containing the two vectors with dCas9 and a non-targeting sgRNA) were included in all experiments and experiments were conducted in four biological replicates in 384-well plates. For each plate, we identified the timepoint when the control strain MM76 (in the presence of erythromycin, chloramphenicol and IPTG at the above-mentioned concentrations) reached plateau, defined as the first timepoint before no increase was detected in log_10_(OD_595_) values of two consecutive timepoints. This timepoint was then used for all wells to derive dose-response curves after normalization to the respective no-drug control for each strain and biological replicate. Full growth curves annotated with the timepoint used for the dose-response curves and dose-response curves with all controls are included in Supplementary Information.

### Determination of cell surface charge

The cytochrome *c* binding assay was conducted as previously described^100^. Briefly, overnight cultures of *S. aureus* Newman were diluted 1:1,000 in 20 ml of TSB medium and grown in flasks at 37 °C with continuous shaking until they reached OD_578_ of ~0.45. Samples were then incubated in the same conditions with or without 10 and 5 µg ml^−1^ ticagrelor for 20 min. Samples were centrifuged at 10,000 *g* for 15 min at room temperature, washed twice with 20 mM MOPS buffer (pH 7) and concentrated to reach a final A_578_ of 10 in a 96-well plate (4483481, Applied Biosystems) containing cytochrome *c* (0.25 mg ml^−1^, 101467, MP Bio) or MOPS buffer (Fig. 5g). The plate was incubated in the dark at room temperature for 10 min. The cell pellets were collected and the amount of cytochrome *c* in the supernatant was determined spectrophotometrically at OD_410_. Two-fold dilutions of cytochrome *c* in the same plate, starting from 256 µg ml^−1^, were used to obtain a standard curve onto which a linear model was fitted to calculate cytochrome *c* concentrations in the other wells. Results are expressed as unbound cytochrome *c* fraction in the supernatants. Experiments were conducted in four biological replicates.

### Reporting summary

Further information on research design is available in the Nature Portfolio Reporting Summary linked to this article.

### Supplementary information

Supplementary Information Supplementary Figs. 1–7.

Reporting Summary

Supplementary Tables **Supplementary Table 1** Strains used in this study. **Supplementary Table 2** Drugs tested in the antibiotic screen. **Supplementary Table 3** Interactions detected in the antibiotic screen. **Supplementary Table 4** Benchmarked interactions from the antibiotic screen. **Supplementary Table 5** Concordance between Loewe and Bliss models on assessing the interactions in the benchmarked set. **Supplementary Table 6** Conserved interactions across Gram-positive and Gram-negative species. **Supplementary Table 7** Known and novel interactions detected in *S. aureus*. **Supplementary Table 8** Number of PBPs and synergies in Gram-positive and Gram-negative species. **Supplementary Table 9** Beta-lactams considered in each Gram-positive or Gram-negative species. **Supplementary Table 10** Correlation between number of PBPs and synergies considering only one strain per species. **Supplementary Table 11** Non-antibiotic drugs tested in *S. aureus* DSM 20231, their therapeutic class and concentration chosen. **Supplementary Table 12** Interactions detected in the non-antibiotic drug screen performed in *S. aureus* DSM 20231. **Supplementary Table 13** Benchmarked interactions from non-antibiotic screen performed in *S. aureus* DSM 20231. **Supplementary Table 14** 2D-TPP hits of ticagrelor treatment of *S. aureus* Newman. **Supplementary Table 15** KEGG annotation of 2D-TPP hits of ticagrelor treatment of *S. aureus* Newman.

Supplementary Data **Supplementary Data 1** Source data for Supplementary Fig. 1, **Supplementary Data 2** Source data for Supplementary Fig. 2, **Supplementary Data 3** Source data for Supplementary Fig. 3, **Supplementary Data 4** Source data for Supplementary Fig. 4, **Supplementary Data 5** Source data for Supplementary Fig. 5, **Supplementary Data 6** Source data for Supplementary Fig. 6, **Supplementary Data 7** Source data for Supplementary Fig. 7.

### Source data

Source Data Fig. 1–5, Source Data Extended Data Fig. 1–9 Statistical source data

## Data Availability

Drug combination data, including raw OD_595_ measurements and growth rates, are available on GitHub at https://github.com/vladchimescu/comBact. An interactive interface to navigate the screen data is available at https://apps.embl.de/combact/. The mass spectrometry proteomics data have been deposited to the ProteomeXchange Consortium via the PRIDE partner repository with the dataset identifier PXD036188. Source data are provided with this paper.

## Code availability

The computational pipeline used to analyse the screen data is available on GitHub at https://github.com/vladchimescu/comBact.
