# Supplementary material for: Systematic analysis of drug combinations against Gram-positive bacteria
Source: Nat Microbiol. 2023 Sep 28;8(11):2196–212. doi: 10.1038/s41564-023-01486-9 (PMC10627819; doi:10.1038/s41564-023-01486-9)
Supplement: Supplementary file 1 — Supplementary Figs. 1–7. [file 41564_2023_1486_MOESM1_ESM.pdf]

nature microbiology

Resource

https://doi.org/10.1038/s41564-023-01486-9

Systematic analysis of drug combinations against Gram-positive bacteria

In the format provided by the authors and unedited

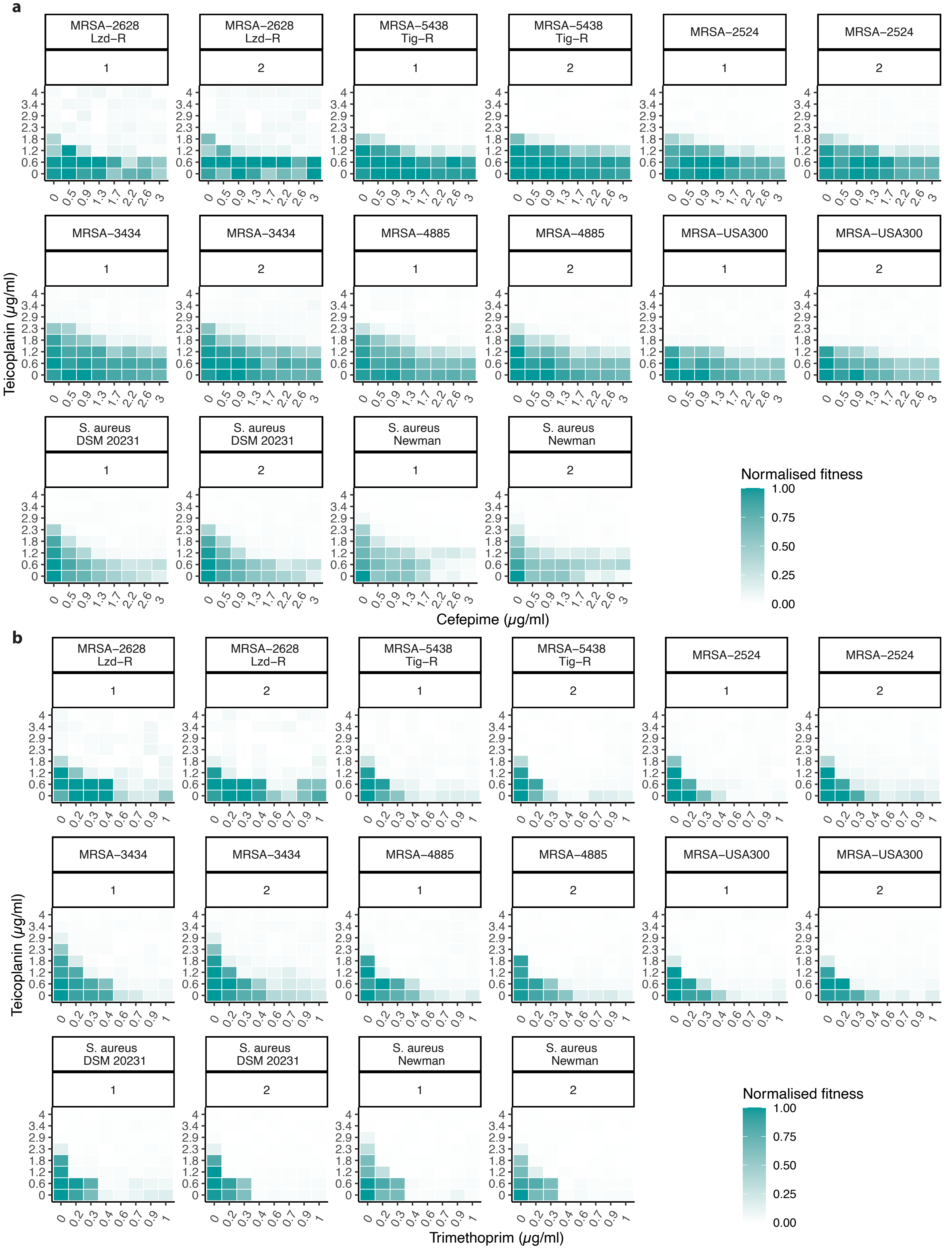

Supplementary Figure 1. Two biological replicates of checkerboard assays displayed in Fig. 2d (synergy between teicoplanin and cefepime (**a**) and between teicoplanin and trimethoprim (**b**), including all strains tested (the model MSSA Newman and DSM 20231 and MRSA USA300 strains, and 5 clinical MRSA strains from different clonal complexes, isolated from different infection sites, with different resistance profiles (Supplementary Table 1, Lzd-R, linezolid-resistant; Tig-R, tigecycline-resistant). Results are obtained and represented as in Fig. 2d.

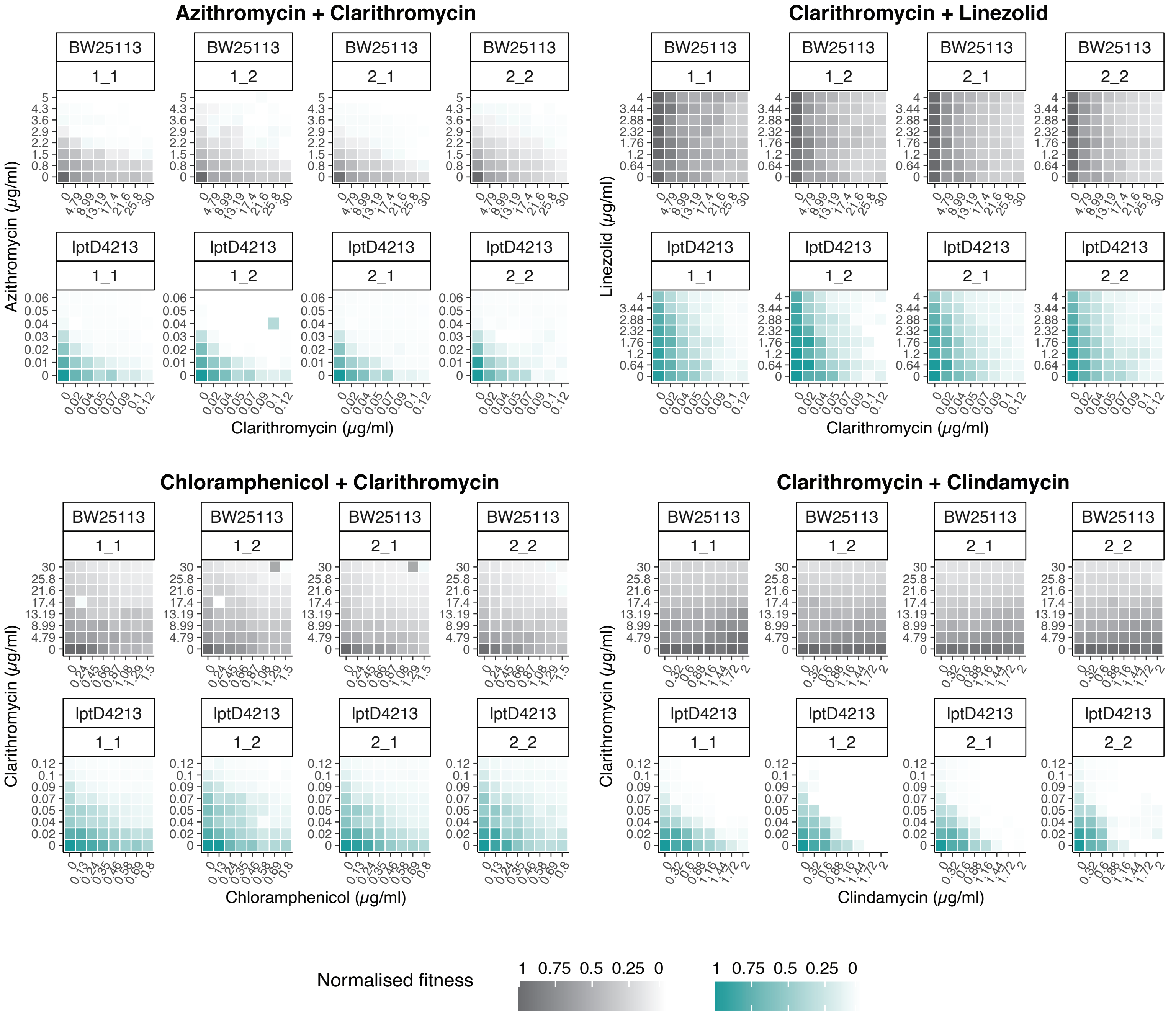

Supplementary Figure 2. Checkerboards from which Bliss interaction scores shown in Fig. 3c were derived. Combinations were tested in each strain (the wild-type *E. coli* BW25113 and the OM-defective *E.coli* lptD4213 strain) in two biological and two technical replicates. Synergy, green; Neutrality, grey. Results are obtained and represented as in Figure 2d.

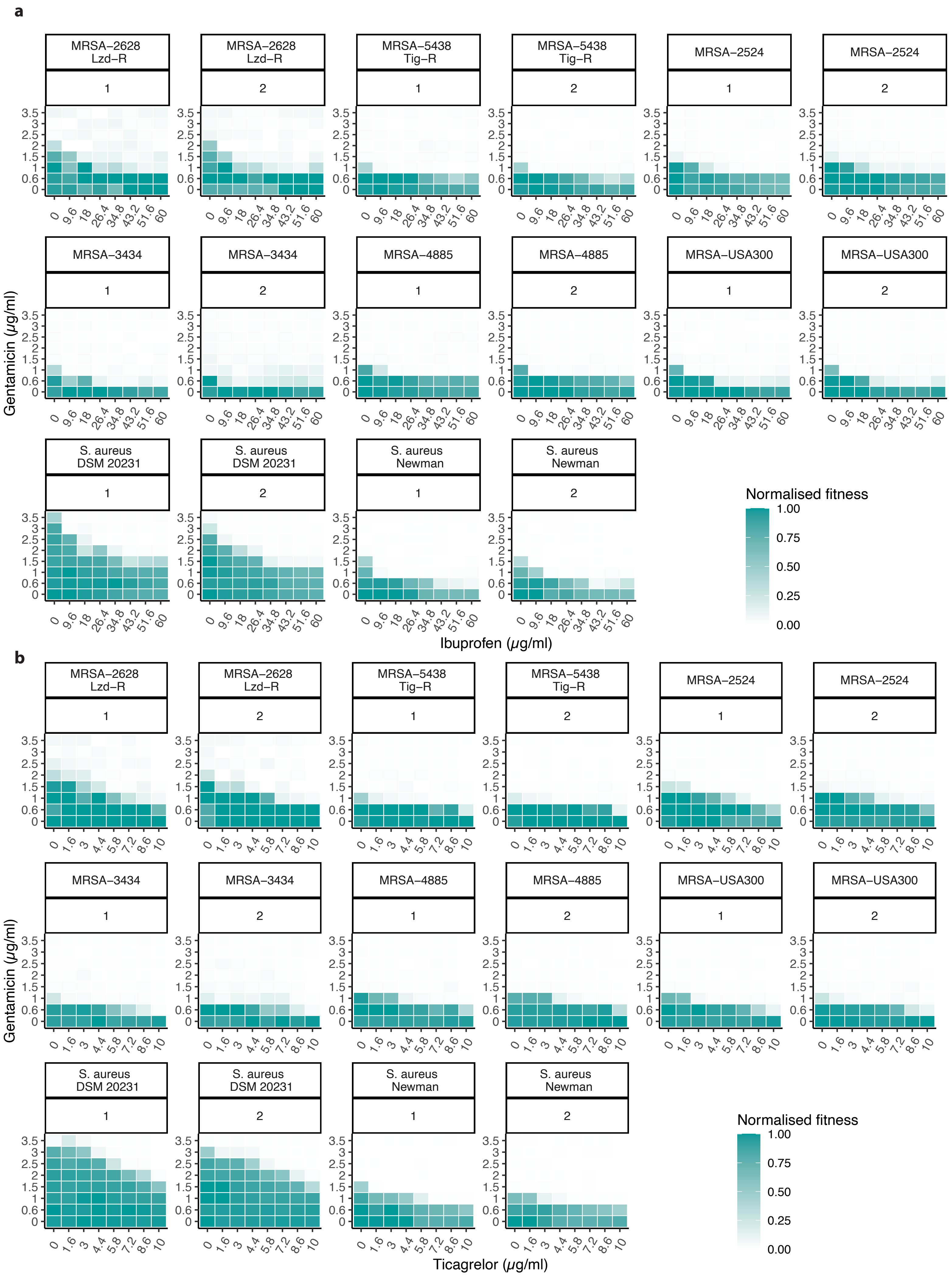

Supplementary Figure 3. Two biological replicates of checkerboard assays displayed in Fig. 4e (**a**) and Fig. 5c (**b**), including all strains tested (the model MSSA Newman and DSM 20231 and MRSA USA300 strains, and 5 clinical MRSA strains from different clonal complexes, isolated from different infection sites, with different resistance profiles (Supplementary Table 1, Lzd-R, linezolid-resistant; Tig-R, tigecycline-resistant). Results are obtained and represented as in Fig. 2d.

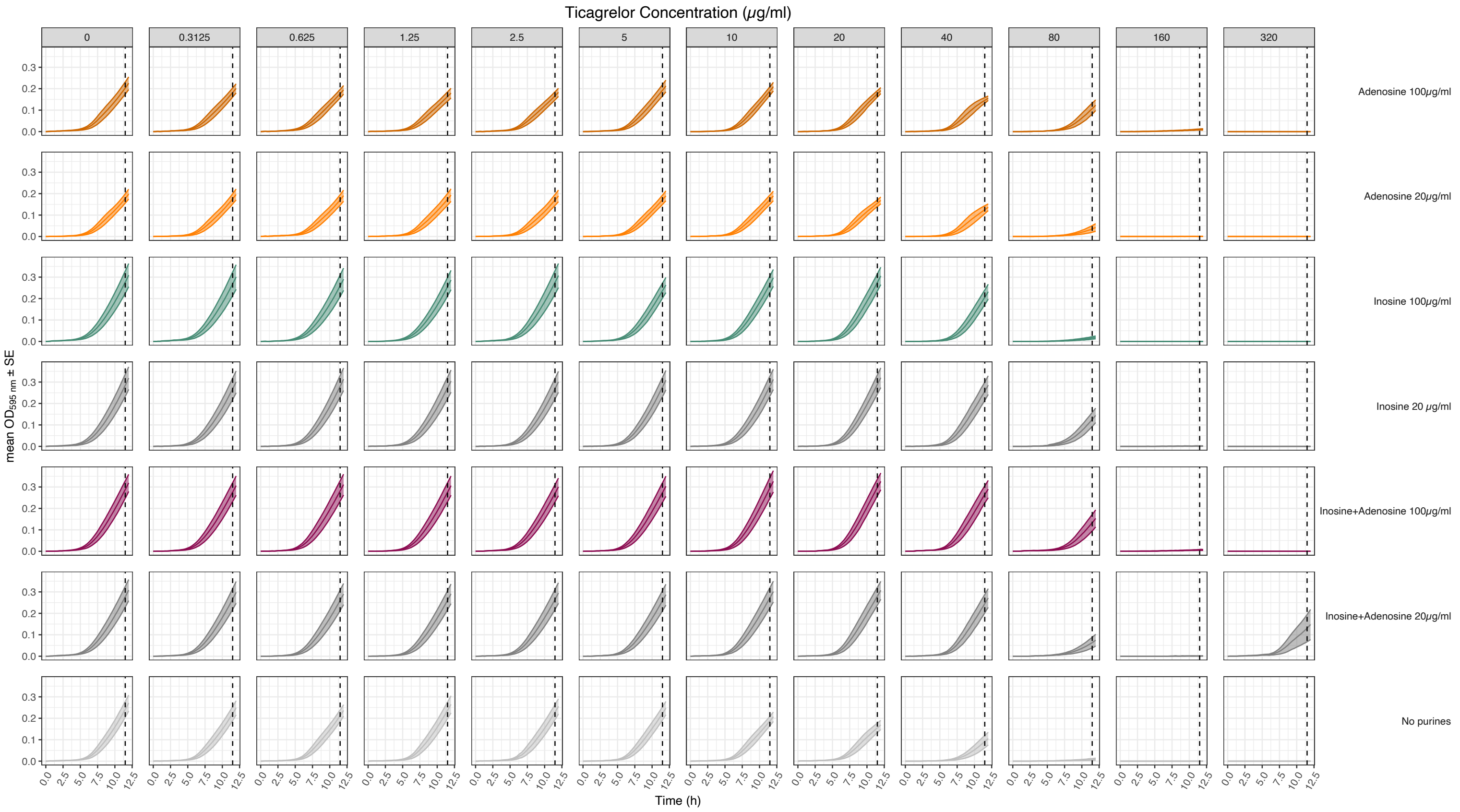

Supplementary Figure 4. Full growth curves from which dose-response curves depicted in Extended Data Fig. 10d were obtained. Mean OD_{595nm} and standard error across four biological replicates are shown. Dashed lines represent the time point used to obtain the dose-response curves.

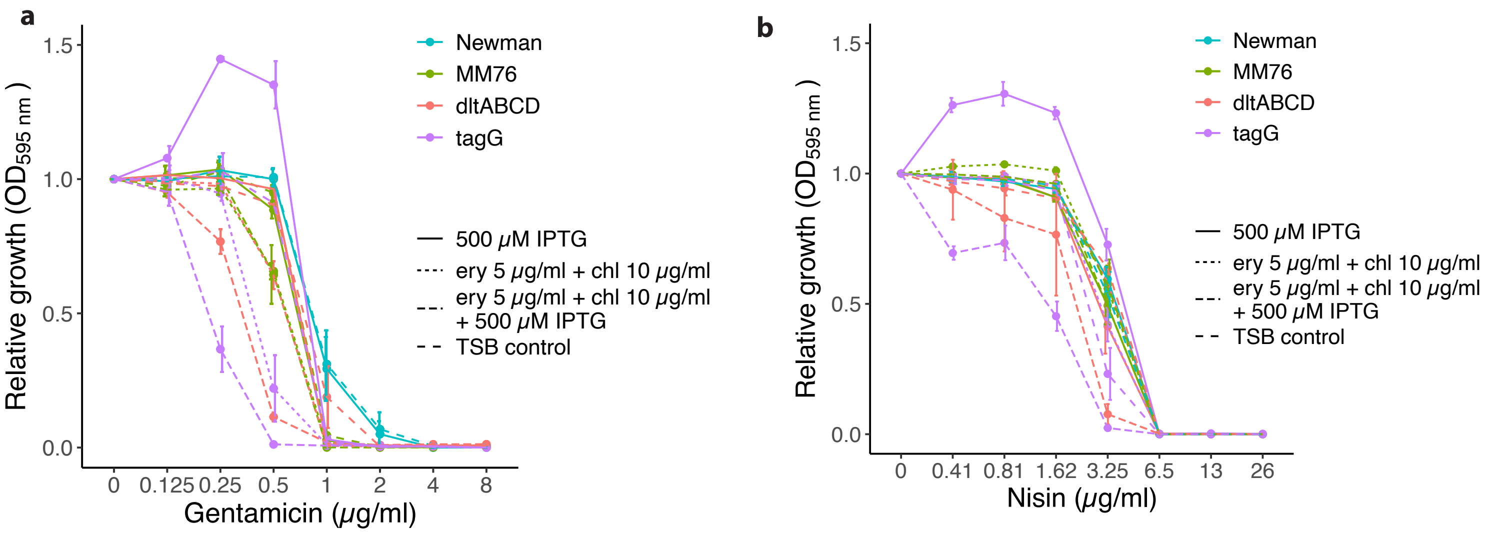

Supplementary Figure 5. Dose-response curves depicted in Fig. 5f (**a**) and Extended Data Fig. 10g (**b**) complete of all controls. Mean OD_{595nm} and standard error across four biological replicates are shown. Ery, erythromycin; chl, chloramphenicol; TSB, Tryptic Soy Broth.

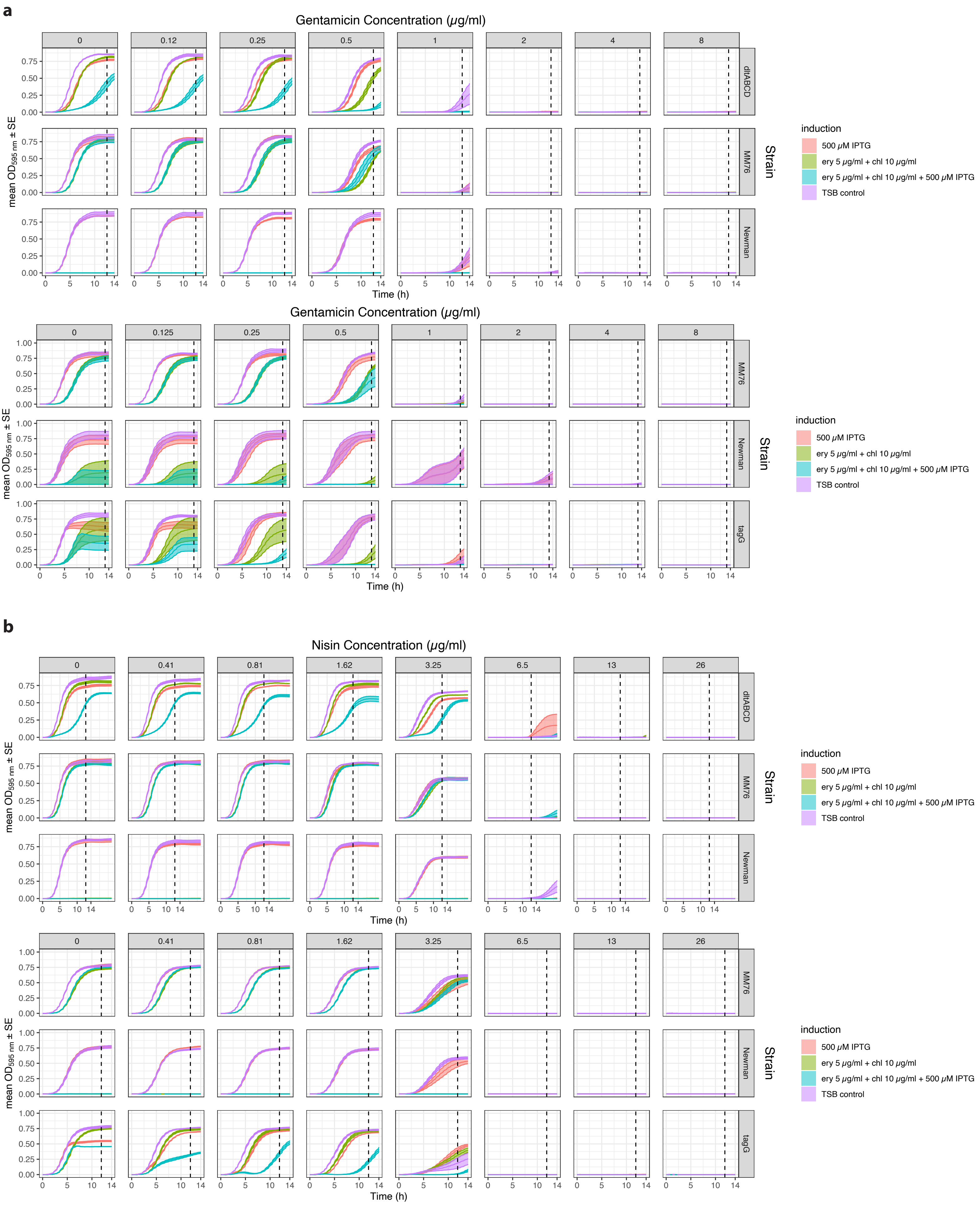

Supplementary Figure 6. Full growth curves from which dose-response curves depicted in Fig. 5f (**a**) and Extended Data Fig. 10g (**b**) were obtained. Mean OD_{595nm} and standard error across four biological replicates are shown. Dashed lines represent the selected time point of OD_{595nm} plateau for the control strain MM76 upon full induction, which was used to obtain the dose-response curves for each experiment (Methods). Ery, erythromycin; chl, chloramphenicol; TSB, Tryptic Soy Broth.

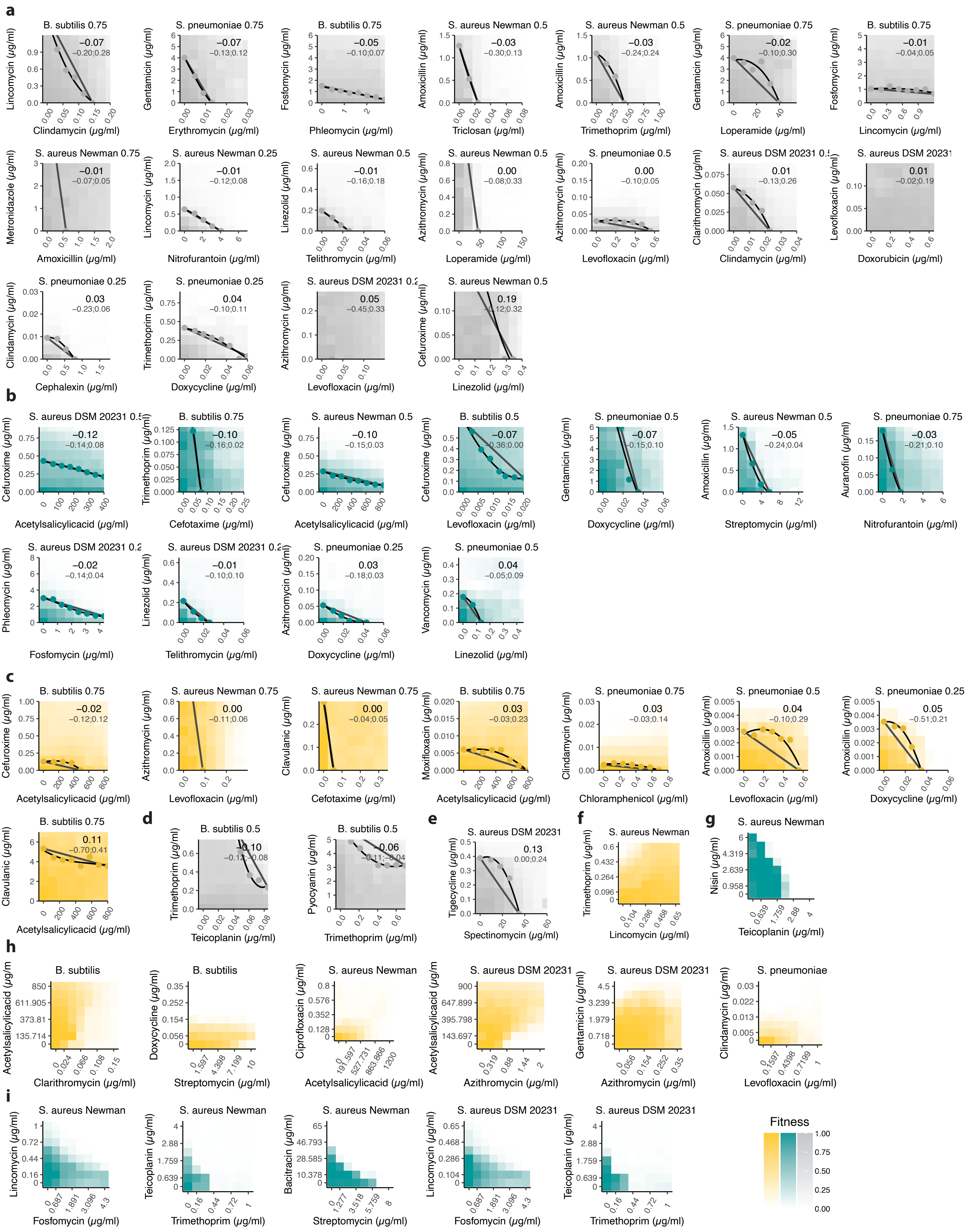

Supplementary Figure 7. Checkerboards from benchmarking set including neutral interactions according to both Loewe and Bliss models (**a**); synergies (**b**) and antagonisms (**c**) only captured by Bliss; synergies (**d**) and antagonisms (**e**) only captured by Loewe model; examples of antagonism (**f**) and synergy (**g**) to which the Loewe model could not be applied; antagonisms (**h**) and synergies (**i**) for which the Loewe model was deemed unreliable (Methods). Results are obtained and represented as in Extended Fig. 4c-d.
