# Supplementary material for: Systematic analysis of drug combinations against Gram-positive bacteria
Source: Nat Microbiol. 2023 Sep 28;8(11):2196–212. doi: 10.1038/s41564-023-01486-9 (PMC10627819; doi:10.1038/s41564-023-01486-9)
Supplement: Supplementary file 2 — Reporting Summary [file 41564_2023_1486_MOESM2_ESM.pdf]

nature portfolio

Corresponding author(s): Athanasios Typas
Last updated by author(s): Aug 10, 2023

Reporting Summary

Nature Portfolio wishes to improve the reproducibility of the work that we publish. This form provides structure for consistency and transparency in reporting. For further information on Nature Portfolio policies, see our Editorial Policies and the Editorial Policy Checklist.

Statistics

For all statistical analyses, confirm that the following items are present in the figure legend, table legend, main text, or Methods section.

n/a	Confirmed	
☐	☒	The exact sample size (n) for each experimental group/condition, given as a discrete number and unit of measurement
☐	☒	A statement on whether measurements were taken from distinct samples or whether the same sample was measured repeatedly
☐	☒	The statistical test(s) used AND whether they are one- or two-sided<br>*Only common tests should be described solely by name; describe more complex techniques in the Methods section.*
☐	☒	A description of all covariates tested
☐	☒	A description of any assumptions or corrections, such as tests of normality and adjustment for multiple comparisons
☐	☒	A full description of the statistical parameters including central tendency (e.g. means) or other basic estimates (e.g. regression coefficient) AND variation (e.g. standard deviation) or associated estimates of uncertainty (e.g. confidence intervals)
☐	☒	For null hypothesis testing, the test statistic (e.g. F, t, r) with confidence intervals, effect sizes, degrees of freedom and P value noted<br>*Give P values as exact values whenever suitable.*
☒	☐	For Bayesian analysis, information on the choice of priors and Markov chain Monte Carlo settings
☒	☐	For hierarchical and complex designs, identification of the appropriate level for tests and full reporting of outcomes
☐	☒	Estimates of effect sizes (e.g. Cohen's d, Pearson's r), indicating how they were calculated

Our web collection on statistics for biologists contains articles on many of the points above.

Software and code

Policy information about availability of computer code

Data collection	SoftMax Pro 7.1 GxP Software; BioTek Gen5 Data Analysis Software v.3.02.2. Proteomics raw data files were processed using isobarQuant (https.://github.com/protcode/isob), Mascot (v. 2.4, Matrix Science).
Data analysis	R v.4.1.2; R packages drm (v0.5.8), BIGL (v1.8.0), TPP2D (v1.14.0), KEGGREST (v1.41.0), EnrichmentBrowser (v2.30.1), clusterProfiler (v3.10.1); Bioconductor (v3.16), Cytoscape v3.8.2, fetchMG v1.0, muscle v3.8.1551, PhymL 3.0. Custom code is available on Github: https://github.com/vladchimescu/comBact. An interactive interface to navigate the screen data is available (https://apps.embl.de/combact/).

For manuscripts utilizing custom algorithms or software that are central to the research but not yet described in published literature, software must be made available to editors and reviewers. We strongly encourage code deposition in a community repository (e.g. GitHub). See the Nature Portfolio guidelines for submitting code & software for further information.

Data

Policy information about availability of data

All manuscripts must include a data availability statement. This statement should provide the following information, where applicable:

- Accession codes, unique identifiers, or web links for publicly available datasets
- A description of any restrictions on data availability
- For clinical datasets or third party data, please ensure that the statement adheres to our policy

Raw drug combination data and the analysis pipeline are available on Github: https://github.com/vladchimescu/comBact. An interactive interface to navigate raw

and processed data is available (https://apps.embl.de/combact/). Detected drug interactions can be found in Supplementary Table 2 and 6. The mass spectrometry proteomics data have been deposited to the ProteomeXchange Consortium via the PRIDE partner repository with the dataset identifier PXD036188. Hits from TPP (Thermal Proteome Profiling) can be found in Supplementary Table 7.

Human research participants

Policy information about studies involving human research participants and Sex and Gender in Research.

Reporting on sex and gender	N/A
Population characteristics	N/A
Recruitment	N/A
Ethics oversight	N/A

Note that full information on the approval of the study protocol must also be provided in the manuscript.

Field-specific reporting

Please select the one below that is the best fit for your research. If you are not sure, read the appropriate sections before making your selection.

☒ Life sciences ☐ Behavioural & social sciences ☐ Ecological, evolutionary & environmental sciences

For a reference copy of the document with all sections, see nature.com/documents/nr-reporting-summary-flat.pdf

Life sciences study design

All studies must disclose on these points even when the disclosure is negative.

Sample size	For all experiments, no prior assumptions were made regarding sample size. Experiments were performed in at least two biological (independent overnight cultures) and two technical replicates. For number of replicates for each experiment, please see figure legends and "Methods".
Data exclusions	Plates with technical replicate correlation (Pearson correlation < 0.7) were excluded from further analysis (for details please see "Methods").
Replication	Experiments were always conducted in at least two biological (independent overnight cultures) and two technical replicates. For number of replicates for each experiment, please see figure legends and "Methods".
Randomization	Randomization was not relevant to our study. Controls were always processed in parallel.
Blinding	For in vitro experiments, no blinding was performed, since it was necessary to keep track of which samples are used in each experiments. Controls were always processed in parallel.

Reporting for specific materials, systems and methods

We require information from authors about some types of materials, experimental systems and methods used in many studies. Here, indicate whether each material, system or method listed is relevant to your study. If you are not sure if a list item applies to your research, read the appropriate section before selecting a response.

Materials & experimental systems

n/a	Involved in the study	
☒	☐	Antibodies
☒	☐	Eukaryotic cell lines
☒	☐	Palaeontology and archaeology
☐	☒	Animals and other organisms
☒	☐	Clinical data
☒	☐	Dual use research of concern

Methods

n/a	Involved in the study	
☒	☐	ChIP-seq
☒	☐	Flow cytometry
☒	☐	MRI-based neuroimaging

Animals and other research organisms

Policy information about studies involving animals; ARRIVE guidelines recommended for reporting animal research, and Sex and Gender in Research

Laboratory animals	The experiments were performed using larvae of the insect lepidopter Galleria mellonella, purchased from UK Waxworms (Sheffield, UK) and Mucha Terra (Ahaus-Altstätte, Germany).
Wild animals	N/A
Reporting on sex	N/A
Field-collected samples	N/A
Ethics oversight	Insect larvae are not subject to ethical regulation.

Note that full information on the approval of the study protocol must also be provided in the manuscript.
